# Comprehensive Structural and Interfacial Characterization of Laser-Sliced SiC Wafers

**DOI:** 10.3390/ma18245615

**Published:** 2025-12-14

**Authors:** Hong Chen, Seul Lee, Minseung Kang, Hye Seon Youn, Seongwon Go, Eunsook Kang, Chae-Ryong Cho

**Affiliations:** 1 Department of Nano Fusion Technology, Pusan National University, Busan 46241, Republic of Korea; chenhong4819@163.com (H.C.); seulthink@pusan.ac.kr (S.L.); twomin0000@naver.com (M.K.); yoonhyeseon0723@naver.com (H.S.Y.); gomoti5711@naver.com (S.G.); 2 R&D Center, Unis Co., Ltd., 1394 Nakdong-daero, Sasang-gu, Busan 46907, Republic of Korea; kes@uniss.asia; 3 Department of Nano Energy Engineering, Pusan National University, Busan 46241, Republic of Korea

**Keywords:** SiC wafer, laser slicing, ARXPS, SIMS, TEM, surface damage, crystal integrity

## Abstract

Laser slicing has emerged as a promising low-kerf and low-damage technique for SiC wafer fabrication; however, its effects on the crystal integrity, near-surface modification, and charge-transport properties require further clarification. Here, a heavily N-doped 4° off-axis 4H-SiC wafer was sliced using an ultraviolet (UV) picosecond laser, and both laser-irradiated and laser-sliced surfaces were comprehensively characterized. X-ray diffraction and pole figure measurements confirmed that the 4H stacking sequence and macroscopic crystal orientation were preserved after slicing. Raman spectroscopy, including analysis of the folded transverse-optical and longitudinal-optical phonon–plasmon coupled modes, enabled dielectric function fitting and determination of the plasmon frequency, yielding a free-carrier concentration of ~3.1 × 10^18^ cm^−3^. Hall measurements provided consistent carrier density, mobility, and resistivity, demonstrating that the laser slicing process did not degrade bulk electrical properties. Multi-scale Atomic Force Microscopy (AFM), Angle-Resolved X-Ray Photoelectron Spectroscopy (ARXPS), Secondary Ion Mass Spectrometry (SIMS), and Transmission Electron Microscopy (TEM)/Selected Area Electron Diffraction (SAED) analyses revealed the formation of a near-surface thin amorphous/polycrystalline modified layer and an oxygen-rich region, with significantly increased roughness and thicker modified layers on the hilly regions of the sliced surface. These results indicate that UV laser slicing maintains the intrinsic crystalline and electrical properties of 4H-SiC while introducing localized nanoscale surface damage that must be minimized by optimizing the slicing parameters and the subsequent surface-finishing processes.

## 1. Introduction

Silicon carbide (SiC) is a third-generation wide-bandgap semiconductor that has become the substrate of choice for power devices in applications such as electric vehicle (EV) main-drive inverters [1], fast charging grid-connected photovoltaics [2], and electric power transmission [3], owing to its high breakdown electric field, high thermal conductivity, and high carrier saturation drift velocity [4]. With the expansion of global power-electronics capacity and rapid rise in automotive demand, there is an urgent need for SiC wafer-manufacturing technologies that deliver high yield, low cost, and low loss [5,6,7,8].

Conventional slicing methods, such as diamond wire sawing, are prone to wafer fracture [9], kerf loss [10,11], surface damage (scratches and microcracks), and subsurface damage [12]. This leads to wastage of expensive raw materials and necessitates time-consuming, labor-intensive post-polishing to meet epitaxial-growth surface requirements, thereby further increasing the overall wafer cost. Compared with traditional diamond-wire cutting, laser slicing has emerged as a leading alternative for SiC wafer fabrication due to its potential for reduced kerf loss, thin-wafer capability, and suitability for hard and brittle materials [13,14,15,16,17]. Laser slicing utilizes high-intensity laser pulses interacting with the ingot, inducing phase separation, point defects, dislocations, and microcracks, and forming a thin damaged layer. The subsequent application of mechanical, thermal, or volumetric expansion stress triggers interlayer delamination [18], thereby separating the damaged layer from the intact single-crystal region.

The machining performance of laser slicing is closely controlled by laser parameters such as wavelength, pulse duration, single-pulse energy/fluence, repetition rate/burst structure, polarization, beam profile, and numerical aperture/aberrations, as well as process-path parameters such as scan speed, pitch/step-over, and depth [19,20,21,22,23]. Together, these factors determine in-bulk absorption, modification thresholds, continuity of the modified layer, and crack propagation paths. In recent years, many studies have focused on expansion and optimization of the process windows. Zhang et al. used ultrafast lasers to create internal modified layers for wafer slicing and systematically examined the effects of pulse duration, pulse energy, and scan speed/spacing/depth on the modified layer structure and exfoliation quality [24]. Combining a weighted Gerchberg–Saxton (GSW) algorithm with spherical aberration correction, Lu et al. generated dual-focus beams to achieve synchronous dual-layer cleaving in SiC, markedly improving the slicing efficiency [25]. Yan et al. optimized the total energy and the number of sub-pulses in temporally shaped ultrafast pulse trains to precisely regulate carrier excitation near the focus, achieving a single modified layer with a thickness of only 16.5 μm and controlled cracking in semi-insulating SiC [26]. Liu et al. proposed an all-laser two-step method for slicing 4H-SiC, in which high-fluence pulses pre-seed microcracks that are subsequently promoted and interconnected by low-fluence pulses, thereby enabling wafer separation with low kerf loss and low surface roughness [27].

In addition to internal laser slicing, various laser-assisted machining routes have been explored to improve the machinability and surface integrity of SiC. Liu et al. investigated reaction-bonded SiC (RB-SiC) by combining laser-assisted cutting experiments with molecular-dynamics nano-cutting simulations over a range of temperatures to examine the property mismatch at the Si/SiC phase boundaries [28]. Their results show that continuous-wave laser heating enhances the ductile response of both phases and suppresses SiC cluster adhesion and elastic recovery at the interfaces, while high-temperature cutting promotes annealing and recrystallization of the disordered phase, thereby reducing residual stress and alleviating cutting-induced damage. On dense SiC ceramics, Zheng et al. employed high-frequency femtosecond-laser polishing and demonstrated, through simulations and experiments, that using a small oblique incidence angle together with optimized pulse energy, repetition rate, and scanning speed suppresses light trapping in surface pits, reduces oxidation and graphitization, and yields pit-free surfaces with surface roughness of S_a_ ≈ 0.187 μm [29]. These laser-assisted cutting and ultrafast-laser polishing approaches highlight the potential of tailored laser processing to enhance SiC surface quality and provide important context for the internal laser slicing strategy investigated in the present work.

Localized laser focusing generates extreme temperature/pressure and ultrafast nonequilibrium dynamics, introducing lattice disorder [30], subsurface defects [31,32,33], and chemical reconfiguration [34,35,36] in the near-surface/subsurface region of SiC. These changes can lead to deleterious effects on the subsequent chemical–mechanical polishing (CMP), epitaxial growth, device integrity, and electrical performance. Previous studies using scanning electron microscopy, transmission electron microscopy/selected area electron diffraction [37,38,39], Raman spectroscopy [40,41,42], X-ray diffraction (XRD) [43], atomic force microscopy (AFM) [44] and X-ray photoelectron spectroscopy (XPS) [45] have observed amorphous/nanocrystalline layers of varying thickness and microcrack networks adjacent to the laser-modified region, accompanied by surface/near-surface oxidation, adventitious contamination, or valence-state rearrangement. These structural and chemical nonidealities require post-processing for repair and passivation [46]. Plasma-based remediation, high-temperature hydrogen etching (hydrogen anneal), sacrificial oxidation followed by oxide removal, and fine CMP have all been shown to reduce subsurface damage, improve roughness, and lower the interface state density, thereby providing a cleaner surface for the subsequent epitaxy and gate dielectric formation [47,48,49]. Recent work further suggests that multi-step plasma schemes [50] can synergistically heal residual defects remaining after CMP, while earlier studies have demonstrated that hydrogen etching [51,52] and sacrificial oxidation [53,54,55,56] can controllably tune the surface morphology and composition of 4H-SiC, providing guidance for post-separation surface/interface reconditioning after laser slicing.

The microstructural evolution, chemical composition gradients, and their associated physical properties at laser-separated surfaces are not fully understood; however, a detailed in-depth understanding of these surface characteristics is essential for assessing the suitability of laser slicing for large-scale manufacturing and for the design of effective post-processing repair strategies. To address this gap, this study conducted a systematic investigation of the surface modifications of SiC wafers after laser slicing. Using a suite of complementary techniques, we directly compared the original polished and laser-separated faces. By integrating XRD, micro-Raman spectroscopy, angle-resolved XPS (ARXPS), secondary ion mass spectrometry (SIMS), scanning electron microscopy (SEM), AFM, four-point-probe electrical measurements, and cross-sectional transmission electron microscopy (TEM), we performed analyses that spanned the length scales from the atomic to the micron scale. We quantitatively evaluated laser-induced differences in the structural disorder, subsurface defects, chemical valence states, and surface roughness, elucidated their implications for electrical performance and interfacial quality, and discussed viable post-treatment pathways for enabling high-quality SiC wafer manufacturing.

## 2. Materials and Methods

### 2.1. Laser System

A pulsed ultraviolet (UV) laser system with a wavelength of 365 nm was employed for the slicing experiments. The laser was operated with a pulse duration (τ) of 10 ps and a repetition rate (f) of 100 kHz, allowing precise control of energy delivery. Each pulse had an energy (E) of 0.30 μJ and was focused to a Gaussian spot with a 1/e^2^ intensity diameter of approximately 8 μm, the diameter of the circle at which the beam intensity drops to 1/e^2^ (≈13.5%) of its on-axis maximum, following the standard definition of a Gaussian beam waist. The corresponding peak intensity and fluence were 1.2 × 10^2^ GW cm^−2^ and 1.2 J cm^−2^, respectively, as calibrated using a power meter and the knife-edge method. During the processing, the laser beam was scanned at a speed (v) of 40 mm s^−1^. The laser polarization was linear and oriented at 90° relative to the scan direction, and all experiments were conducted under nitrogen-purged atmosphere (flow rate: 5–10 L min^−1^) to suppress oxidation during irradiation. A comparison of the laser parameters used in this study with those reported in previous studies is provided in Table S1, illustrating the similarities and differences in our experimental conditions compared to those used in similar slicing experiments. These laser parameters were chosen within the process windows reported for 4H-SiC laser slicing and summarized in Table S1. UV wavelength (365 nm) and 10 ps pulses at 100 kHz are standard for commercial slicing tools. A pulse energy of 0.30 μJ focused to an 8 μm (1/e^2^) spot gives a fluence slightly above the modification threshold without causing catastrophic cracking. The scan speed of 40 mm·s^−1^ ensures strong pulse overlap and a continuous buried damage layer. The scan direction was aligned with the 4° off-cut direction of the wafers to represent the typical slicing configuration of the supplied off-axis 4H-SiC substrates. A schematic of the laser system is provided in Figure S1.

### 2.2. Materials and Characterization

The starting N-doped 4H-SiC material is a 4° off-axis n-type single-crystal substrate with a nitrogen doping concentration of 1 × 10^18^ cm^−3^. Single-crystalline 4H-SiC ingots were subjected to laser slicing using a pulsed ultraviolet laser system (λ = 365 nm) with a controlled pulse width and repetition rate to achieve precise separation. The resulting wafer (~120 μm thick) was divided into front (laser-irradiated) and back (laser-sliced) regions for comparison. XRD, micro-Raman spectroscopy, SEM/Energy-Dispersive X-ray Spectroscopy (EDS), AFM, ARXPS, SIMS, and four-point probe measurements, and focused ion beam (FIB)-TEM/EDS analyses were performed as previously described. A JEOL JSM-IT800 (JEOL, Akishima, Tokyo) scanning electron microscope (operating conditions: accelerating voltage: 15.0 kV, probe current: 5 nA, working distance: 10 mm) was used for SEM to observe microscale surface features such as the recast layer, spatter, edge burrs, and microcracks. High-resolution secondary electron images were also acquired. EDS was performed using an energy-dispersive X-ray spectrometer integrated into the above scanning electron microscope (energy resolution 127 eV at 130,000 cps) for elemental point analyses and area mapping to obtain the semi-quantitative atomic percentages of Si, C, O, and N (operating conditions: accelerating voltage: 15.0 kV, probe current: 5 nA, working distance: 10 mm). A Seiko Instruments SPA-400 atomic force microscope (Seiko Instruments, Chiba, Japan) operating in the tapping mode (lateral resolution: 0.2 nm, vertical resolution: 0.01 nm, scan range: XY 20 µm, Z 1 µm) was employed to scan the surface and obtain the three-dimensional topography as well as the quantitative roughness parameters (arithmetic mean roughness (R_a_) and root-mean-square roughness (R_rms_)) for assessing the nanoscale undulations induced by laser processing. The XRD measurements were performed using an XPERT-PRO diffractometer (goniometer PW3050/65) (PANalytical B.V., Almelo, The Netherlands) with monochromated Cu Kα1 radiation (λ = 1.5406 Å). XRD was used to identify the bulk crystal structure and detect new phases potentially induced by laser machining. Raman spectra were acquired under 532 nm excitation using a Raman spectrometer (Horiba, LabRam, HR 800, Kyoto, Japan) to characterize the laser-induced disordered/graphitic-like carbon, and the defect density was quantified from the D/G intensity ratio (I_D_/I_G_). 20 × 20 µm**^2^** maps were acquired for both the laser-irradiated and laser-sliced surfaces, and in addition to the carbon bands at ~1350 and ~1580 cm^−1^, the 4H-SiC folded transverse optic (FTO(1/2)) mode at ~777 cm^−1^ was mapped to assess the substrate crystallinity. To analyze the laser-sliced SiC damage layer, recast layer, and their nanoscale structure and local chemistry relative to the polished surface, cross-sectional lamellae were prepared by FIB milling using a Thermo Scientific Helios 5 UX system (Thermo Scientific, Waltham, MA, USA) (operating conditions: 3 kV, 0.40 nA probe current, 12,000× magnification, working distance: 4.0 mm, horizontal field width: 10.6 µm) and subjected to combined TEM (Thermo Fisher Talos F200X, Thermo Scientific, Waltham, MA, USA), SAED, and EDS measurements. TEM imaging was performed at 200 kV in the High-Angle Annular Dark-Field Scanning Transmission Electron Microscopy (HAADF-STEM) mode. SAED patterns were collected to index plane families and evaluate crystalline and orientation dispersion. The EDS point, line, and area mapping focused on the spatial distributions of Si, C, O, and N across the surface and transition zone.

For nondestructive information about the surface chemical states and their depth distribution, ARXPS measurements were carried out using a Thermo Fisher Theta Probe AR-XPS System (Thermo Scientific, Waltham, MA, USA, monochromated Al K_α_, hv = 1486.6 eV). The operating conditions were 15 kV, 100 W X-ray energy, and a spot size of 400 µm. The photoelectron takeoff angle was varied from 28° to 78° relative to the surface normal to control the effective probing depth. The core-level spectra of C 1s, O 1s, N 1s, and Si 2p were recorded and are given using the C–C signal at 284.6 eV as the reference. The relative peak areas and their angular dependences were used to determine the vertical stratification of the chemical components (such as the C–C, C–Si, C–O, Si–O, and N-containing functionalities). Elemental depth profiling was performed using a CAMECA Magnetic Sector SIMS 7f-auto secondary ion mass spectrometer (CAMECA SAS, Gennevilliers, France) with a 7.5 keV O2+ primary ion beam for sputtering and analysis, using a current of 20 nA and an analysis area of 250 × 250 µm^2^. The detected ions included ^12^C^+^ and ^30^Si^+^. For depth profiling, the same instrument was operated using a Cs + primary ion source at 15.0 keV and 150 nA. A raster size of 150 × 150 μm^2^ was used with an analysis area of 30 μm in diameter, and the detected ions included ^12^C^−^, ^12^C^14^N^−^, ^28^Si^−^, and ^18^O^−^. Hall effect and resistivity measurements were performed at room temperature using an Ecopia HMS 3000 Ver. 3.1 four-point probe system (Ecopia Corporation, Anyang, Gyeonggi-do, Republic of Korea; HMS 3000 software, Ver. 3.1) in accordance with van der Pauw method. The samples were mounted on a spring-clip board, and a constant current of 100 μA was applied while recording the Hall voltage. The bulk and sheet carrier concentrations, electrical conductivity, sheet resistance, electron mobility, and Hall coefficient were determined using the built-in analysis software. Hall-effect and resistivity measurements on our specimen confirm n-type conduction with a high donor concentration, in agreement with the other analysis in this work. XPS and SIMS are mainly used to monitor relative changes in nitrogen-related signals at and near the laser-processed surfaces rather than to re-determine the absolute bulk dopant concentration.

## 3. Results and Discussion

**Figure 1:**
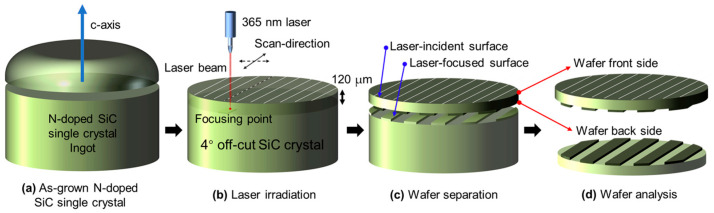
Schematic illustration of the UV laser slicing process for detaching a wafer from a 4° off-cut N-doped 4H-SiC ingot. The 4H-SiC ingot, grown along the c-axis and oriented with a 4° off-cut toward the [11–20] direction to suppress polytype inclusions, is aligned for laser processing. A focused pulsed UV laser (λ = 365 nm) is introduced at a controlled subsurface depth to form a periodic laser-modified layer that serves as a predetermined cleavage plane. Scanning the laser across the wafer area generates localized stress and decomposition along this layer, enabling clean mechanical separation and producing a thin SiC wafer with minimal thermal or structural damage.

The fabrication process of the laser-sliced SiC wafers is illustrated in Figure 1. A high-purity 4H-SiC single-crystal ingot grown via physical vapor transport (PVT) was used as the starting material. The ingot was oriented along the [0001] c-axis and subsequently polished with a 4° off-cut in the [11–20] direction to promote step-flow epitaxial growth and minimize the formation of micropipes or polytype inclusions. A pulsed UV laser (λ = 365 nm, τ ≈ 10 ps) was employed to locally modify the crystal beneath the wafer surface. The laser beam was tightly focused at a predetermined focal depth (~120 µm) inside the SiC bulk. By scanning the laser beam laterally across the surface while maintaining a constant focal plane, a periodic subsurface structure known as a laser-modified layer was formed. The pulse energy and repetition rate were carefully tuned to ensure localized melting or micro-decomposition without macroscopic cracking. The complete set of processing parameters used in this study, together with representative conditions used in previous SiC laser processing studies (covering Ultraviolet to Near-Infrared (UV–NIR) wavelengths, ps–ns pulse widths, different scan strategies, atmospheres, and fluence ranges), are summarized in Table S1, providing the context for the specific processing window realized here. After the laser processing, a mechanical or thermal stress gradient was applied to the irradiated region. The energy concentration and defect accumulation at the laser-modified interface induce cleavage along the processed plane, allowing the wafer to spontaneously separate from the bulk crystal without the need for conventional wire saw slicing. The detached wafer retained the same crystallographic orientation as the parent ingot and exhibited atomically flat terraces corresponding to an off-cut angle of 4°. This method effectively minimizes kerf loss, reduces subsurface damage, and enables high-throughput, high-yield SiC wafer fabrication. Moreover, the laser-slicing process is compatible with the subsequent polishing or epitaxial growth steps, making it a promising approach for next-generation semiconductor manufacturing. For the as-obtained wafer, the surface facing the incoming beam is referred to as the front side of the wafer (laser-irradiated), whereas the surface corresponding to the separation plane is referred to as the back side of the wafer (laser-sliced). These two sides were used for further characterization. Figure S2 shows photographs of the 8-inch N-doped 4H-SiC wafer after laser slicing to further illustrate the distinct characteristics of the laser-irradiated and laser-sliced surfaces. The two sides of the laser-sliced 4H-SiC wafer exhibit distinct morphologies owing to the different mechanisms involved in the laser-based separation process. The laser-irradiated side was directly exposed to the scanning laser during slicing. Localized thermal accumulation and stress concentration produced periodic trace-like surface textures and directional patterns characteristic of laser–matter interactions. Consequently, this surface had a rougher appearance and displayed visible laser-track patterns. By contrast, the laser-sliced side represents the natural fracture plane formed at the focal depth of the ultraviolet laser (~120 μm). Because separation occurs internally without mechanical abrasion, this surface exhibits a relatively smooth morphology with minimal kerf loss and mechanical damage. This comparison clearly demonstrates that the laser-induced internal cracking and surface irradiation have different effects on the final wafer surface characteristics.

### 3.1. Surface Morphology

**Figure 2:**
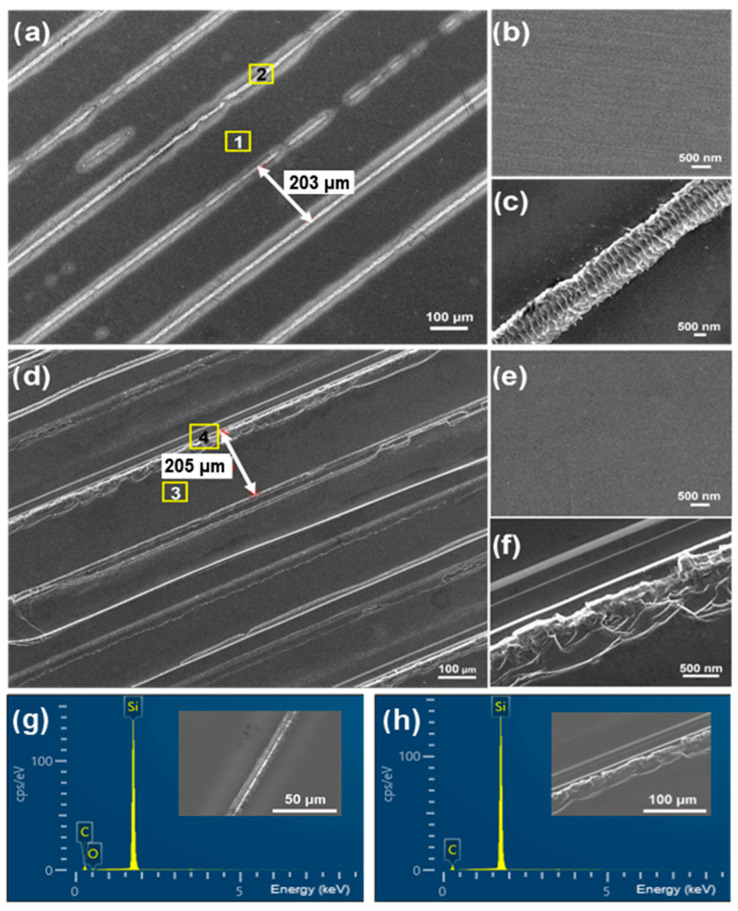
SEM and EDS analyses of the laser-irradiated and laser-sliced SiC surfaces. (**a**) Low-magnification SEM image of the laser-irradiated surface showing periodic laser tracks with a spacing of ~203 μm. (**b**,**c**) High-magnification SEM images of regions 1 and 2 from (**a**), revealing the surface morphology prior to wafer separation. (**d**) Low-magnification SEM image of the laser-sliced surface with a spacing of ~205 μm, exhibiting fracture features produced by subsurface laser modification. (**e**,**f**) High-magnification SEM images of regions 3 and 4 from (**d**), showing the microstructure of the laser-sliced fracture plane. (**g**,**h**) EDS spectra from the laser-irradiated surface (**g**) and the laser-surface (**h**). Inset electron-microscopy images confirm that both surfaces contain only Si and C without detectable contamination.

Figure 2 compares the surface morphologies and EDS results of the laser-irradiated and laser-sliced SiC surfaces, with Figure 2a–f showing results for the laser-irradiated and the laser-sliced sides, respectively. A low-magnification SEM image of the laser-irradiated surface (Figure 2a) shows periodic laser irradiated tracks with a spacing of 203 μm, where each track consists of a relatively smooth irradiated region surrounded by recast ridges. Figure 2b presents a higher-magnification image of region “1” in Figure 2a, revealing excellent flatness with only extremely fine processing traces originating from the polishing process. Figure 2c shows a higher-magnification view of the cutting edge on the laser-irradiated surface (region “2” in Figure 2a); along the groove direction, regular and continuous wrinkle ripple-like protrusions with a uniform pitch are observed, and slight buildup and step features appear at the edges, merging into a single recast ridge. Owing to the high local temperature under laser irradiation, the surface underwent rapid heating, melting, and vaporization decomposition, after which the molten and evaporated material rapidly solidified, creating a recast layer at the cutting edges and across the surface. Figure 2d provides a low-magnification overview of the laser-sliced surface with a cutting-line pitch of 205 μm while Figure 2e,f are higher-magnification images of regions “3” and “4” in Figure 2d, respectively. In Figure 2e, fine textures attributable to thermomechanical loading are evident. As shown in Figure 2f, compared to the laser-irradiated surface, through-thickness voids and cracks, together with lamellar spallation, were observed, and the previously continuous ripples were disrupted. The high-energy density of the laser rapidly heats the cutting region to extremely high temperatures, whereas the diffusion and convection effects near the focal point create a strong temperature gradient within the material. This results in a thermal expansion mismatch between the recast layer and SiC substrate, and the resulting thermal stresses promote the formation and propagation of microcracks at the interface [38]. The SiC wafer was detached, leaving lamellar flakes and voids on the laser-cut surface. EDS analyses were performed for representative regions of the laser-irradiated (Figure 2g) and laser-sliced (Figure 2h) surfaces and in both cases, the spectra were dominated by Si and C from the SiC matrix. The quantified elemental compositions of the two regions are summarized in Table S2. The laser-irradiated surface exhibited a trace O contribution because the mechanical abrasion during polishing exposed numerous unsaturated dangling bonds on the SiC surface [5,57]. These highly reactive sites react with oxygen (originating from O_2_ or H_2_O in the polishing medium or ambient air), thereby forming an increased amount of Si–O bonds. Meanwhile, high temperatures and rapid solidification in the laser-incident region led to oxidation reactions, resulting in the formation of Si–O bonds on the laser-irradiated surface. By contrast, the laser-sliced surface, which detached directly from the interior of the SiC wafer, was exposed to much less oxygen during the cutting process. Due to the limited interaction with oxygen molecules compared with the laser-irradiated region, the formation of oxygen-related components is strongly suppressed for the laser-sliced surface. Table S2 confirms that no significant O content is detected on this side.

### 3.2. Surface Roughness

**Figure 3:**
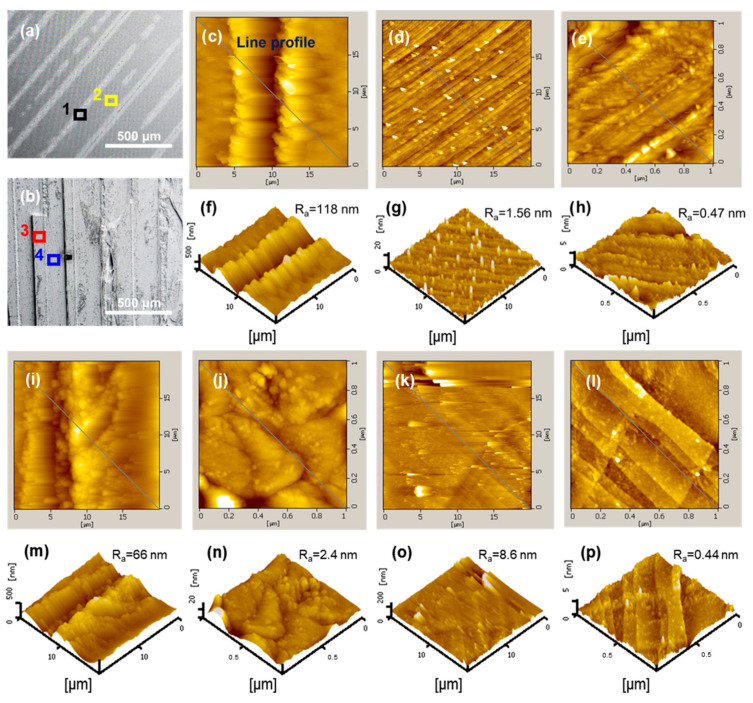
AFM and SEM analyses of the laser-irradiated and laser-sliced SiC surfaces. SEM images of (**a**) the laser-irradiated surface and (**b**) the laser-sliced surface, with the probed AFM regions indicated. AFM 2D morphology and 3D topography were obtained from the laser-irradiated surface at (**c**,**f**) region 1 (20 × 20 μm^2^, black square), (**d**,**g**) region 2 (20 × 20 μm^2^, yellow square), and (**e**,**h**) a 1 × 1 μm^2^ subregion within region 2. For the laser-sliced surface, AFM measurements were collected from (**i**,**m**) region 3 (20 × 20 μm^2^, red square), (**j**,**n**) a 1 × 1 μm^2^ subregion within region 3, (**k**,**o**) region 4 (20 × 20 μm^2^, blue square), and (**l**,**p**) a 1 × 1 μm^2^ subregion within region 4. Regions 1–4 correspond to the laser-irradiated area, smooth area, hill area, and valley area, respectively. Diagonal lines in the AFM images indicate the directions along which surface roughness profiles were extracted.

The surface morphology and roughness of the laser-irradiated and laser-sliced surfaces were analyzed using AFM. The SEM images in Figure 3a,b provide an overview of the surface features of the laser-irradiated and laser-sliced sides, respectively. Figure 3a shows the laser-irradiated surface of the SiC sample, which is relatively flat and uniform, with regions scanned by AFM marked as “1” (black square) and “2” (yellow square). Figure 3b shows the laser-sliced surface, which exhibits more pronounced surface features, including hills and valleys, with regions scanned by AFM marked as “3” (red square, hill) and “4” (blue square, valley). Figure 3c–e presents the AFM 2D morphologies of the laser-irradiated surface in regions 1 and 2, respectively. The scan areas are 20 × 20 μm^2^ for Figure 3c,d and 1 × 1 μm^2^ for Figure 3e. Overall, the 2D maps revealed a relatively flat surface with only small-amplitude undulations. The corresponding 3D topography images are shown in Figure 3f–h. Arithmetic mean roughness (R_a_) and root-mean-square roughness (R_rms_) were extracted from the AFM height data. R_a_ represents the mean absolute deviation from the average height, whereas R_rms_ is more sensitive to large-height excursions owing to its square-averaging definition. In region 1 (20 × 20 μm^2^, Figure 3f), the surface exhibits long-wavelength waviness associated with polishing/processing marks, giving an occasional high average roughness of R_a_ ≈ 118 nm and a root-mean-square roughness of R_rms_ ≈ 162 nm. This large R_rms_ value reflects the presence of larger-scale roughness features (polishing streaks and machining marks), which are more pronounced than the average height deviations captured by R_a_. By contrast, region 2, which is farther from the cutting traces, has a much flatter morphology, with the 20 × 20 μm^2^ scan (Figure 3g) yielding R_a_ ≈ 1.56 nm and R_rms_ ≈ 2.15 nm, while the 1 × 1 μm^2^ scan (Figure 3h) shows only nanoscale height fluctuations with R_a_ ≈ 0.47 nm and R_rms_ ≈ 0.63 nm, indicating that the locally polished terrace still maintains an atomically smooth surface at the sub-micrometer scale. Figure 3i–l shows the AFM 2D morphology of the laser-sliced SiC surface in the hill (region 3) and valley (region 4) areas. The scan areas are also 20 × 20 μm^2^ for Figure 3i,k, and 1 × 1 μm^2^ for Figure 3j,l. The corresponding 3D topography images and roughness values are presented in Figure 3m–p. In the hill region, the 20 × 20 μm^2^ scan (Figure 3m) reveals a strongly corrugated topography composed of ridges and grooves, with R_a_ ≈ 66 nm and R_rms_ ≈ 83 nm, indicating pronounced height fluctuations and a thick recast layer produced by laser cutting. When the scan size is reduced to 1 × 1 μm^2^ (Figure 3n), the surface still shows distinct nanoscale protrusions and depressions, with R_a_ ≈ 8.6 nm and R_rms_ ≈ 3.4 nm, evidencing substantial local melting, rapid resolidification, and microcrack-related roughening. By contrast, the valley region was relatively smoother. For the 20 × 20 μm^2^ scan (Figure 3o), the obtained roughness values are R_a_ ≈ 2.4 nm and R_rms_ ≈ 1.4 nm, while the 1 × 1 μm^2^ scan (Figure 3p) yields R_a_ ≈ 0.44 nm and R_rms_ ≈ 0.56 nm. Although the valleys were rougher than the laser-irradiated smooth surface, their roughness was significantly lower than that of the hill regions, indicating that material removal and crack opening occurred predominantly in the hills, whereas the valleys retained a comparatively flat morphology. The R_a_ and R_rms_ values demonstrate that the laser-irradiated surface maintains a low roughness and uniform surface characteristics, whereas the laser-sliced surface exhibits a highly heterogeneous topography with very rough recast ridges, moderately rough nanoscale hill areas, and relatively smooth valleys. These differences indicate that laser cutting induces strong localized thermal effects, leading to rapid solidification, splatter deposition, and local cracking, which in turn generate irregular surface undulations and uneven roughness. These results highlight that optimization of the laser parameters is necessary to suppress excessive hill formation and microcrack development, and thus improve the cut surface quality.

Figure S3 presents the comprehensive line-profile plots obtained from various regions of the 4H-SiC wafer. The measured areas included (i) the laser-irradiated region, (ii) the non-irradiated region on the same laser-irradiated side, and (iii) the hill and valley regions formed on the laser-sliced side. Each location was scanned at two different magnifications (20 × 20 μm^2^ and 1 × 1 μm^2^) to evaluate both large-scale roughness features and nanoscale surface textures. The height-profile plot corresponding to each AFM image provides quantitative roughness information along with the indicated lateral scan trace. For the laser-irradiated region (20 × 20 μm^2^), the AFM topography of the laser-irradiated region reveals pronounced and periodically aligned surface protrusions produced during the surface scanning of the UV laser. These features correspond to a laser-induced hill–valley texture that arises from localized thermal accumulation, partial remelting, and rapid quenching. The height profile confirms a large vertical variation, with peak-to-valley amplitudes approaching several hundred nanometers, which is consistent with strong laser–matter interactions at the surface. For the non-irradiated region on the laser-irradiated side (20 × 20 μm^2^ and 1 × 1 μm^2^), despite its location on the laser-irradiated side, the non-irradiated region exhibited significantly lower roughness because it was not directly exposed to the scanning beam. The AFM images show a relatively featureless surface with fine asperities, which is typical of polished SiC substrates. The line profiles reveal small height fluctuations on the order of a few nanometers, indicating minimal thermal damage and mechanical disturbance beyond the laser-scanned tracks. The hill regions on the laser-sliced side (20 × 20 μm^2^ and 1 × 1 μm^2^) represent the upper portions of the fracture morphology generated during internal laser-induced cleaving. The AFM topography displayed upwardly elevated features associated with the crack propagation front. The roughness was moderate compared with that of the laser-irradiated surface but notably higher than that of the valley regions. Height profiles indicate peak-to-valley variations, typically ranging from tens to hundreds of nanometers, reflecting local stress-release patterns at the fracture interface. In contrast to the hill regions, the valley regions on the laser-sliced side (20 × 20 μm^2^ and 1 × 1 μm^2^) show a remarkably smoother morphology. This smoother topology corresponds to the natural fracture plane created at the laser focal depth (~120 μm), where internal micro-cracking occurs with minimal thermal input. AFM results reveal small-scale height variations in the 1 × 1 μm^2^ scans that are often below several nanometers. These features confirm that the valley regions retain the intrinsic atomic-scale cleavage characteristics of 4H-SiC with negligible kerf loss and mechanical abrasion.

### 3.3. Structural Phase Transformation

**Figure 4:**
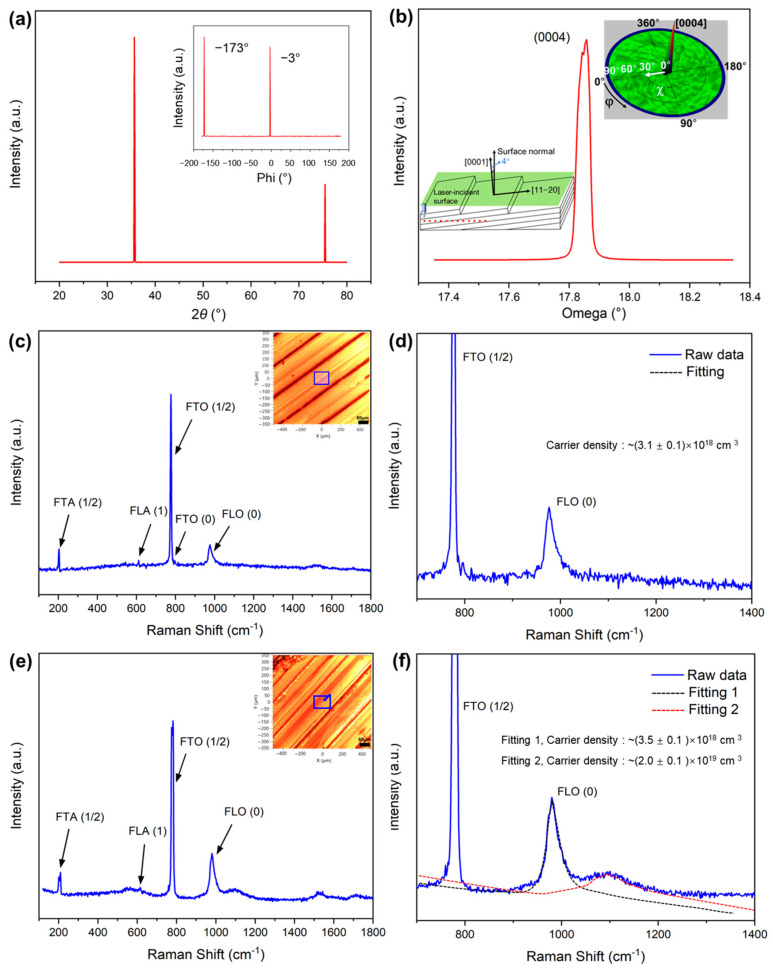
Structural and Raman analyses of the 4H-SiC wafer and laser-irradiated surface. (**a**) XRD θ–2θ scan showing the (0004) and (0008) reflections of 4H-SiC, confirming c-axis–oriented single crystallinity. The inset shows the φ-scan of the (0004) reflection, indicating the absence of in-plane rotational domains. (**b**) Rocking curve (ω-scan) of the (0004) peak with a FWHM of 138 arcsec, demonstrating high crystalline quality and low mosaic spread. The schematic inset illustrates the 4° off-axis geometry toward the [11–20] direction. The (0004) pole figure inset (χ = 0° at the center) exhibits a single sharp intensity maximum corresponding to the c-axis orientation. (**c**) Raman spectra collected from representative regions of the laser-irradiated surface. The inset optical image shows the probed positions. Characteristic folded transverse optical (FTO), folded longitudinal optical (FLO), and folded longitudinal acoustic (FLA) modes of 4H-SiC are identified. (**d**) Enlarged Raman spectra in the 700–1400 cm^−1^ range, highlighting the dominant FTO (1/2) and FLO (0) modes. The FLO (0) peak was fitted using a coupled LO phonon–plasmon model, yielding an electron concentration of approximately (3.1 ± 0.1) × 10^18^ cm^−3^. (**e**) Raman spectra collected from representative regions of the laser-sliced surface. The inset optical image shows the probed positions. (**f**) Enlarged Raman spectra in the 700–1400 cm^−1^ range, highlighting the dominant FTO (1/2) and FLO (0) modes. The FLO (0) peak was fitted using a coupled LO phonon–plasmon model, yielding an electron concentration of approximately (3.5 ± 0.1) × 10^18^ cm^−3^ for fitting 1 and approximately (2.0 ± 0.1) × 10^19^ cm^−3^ for fitting 2.

The diffraction pattern in Figure 4a shows two dominant peaks indexed to the (0004) and (0008) planes, corresponding to the 4H-SiC polytype. No additional peaks (e.g., (10–14), (11–28)) were observed, confirming that the wafer surface was perfectly aligned with the (0001) basal plane and that the growth was epitaxially oriented along the c-axis. The sharpness and intensity of the (0004) reflection indicate a high degree of crystallinity and minimal defect density. The inset in Figure 4a shows the φ-scan around the (0004) plane. A single peak at φ ≈ 0° and −180° indicates the absence of rotational twin or grain misalignment, confirming single-domain orientation over the measured area. This result suggests that the wafer surface was well-polished and that the off-axis cut did not introduce noticeable in-plane distortions. The ω-scan of the (0004) reflection in Figure 4b reveals a narrow FWHM of 138 arcsec (≈0.038°). This small rocking width indicates a low mosaicity, implying that the lattice planes were nearly parallel to the X-ray-illuminated region. Such a narrow width is a characteristic of high-quality single-crystal 4H-SiC substrates that are typically used for epitaxial growth and laser-slicing experiments. The inset pole figure in Figure 4b shows a single-intensity maximum at x = 0°, which is consistent with a perfect (0001) orientation. No secondary lobes or ring-shaped features are observed, confirming the absence of tilted grains or misoriented domains. The rocking curve FWHM (Δω = ±0.038°) corresponds to the angular width of the intensity distribution in the pole figure, quantitatively linking the ω-scan mosaic spread and the χ-tilt distribution.

The Raman spectrum (Figure 4c) of the 4H-SiC crystal clearly reveals both the zone-folded phonon modes characteristic of the polytype structure and the longitudinal optical phonon–plasmon coupled (LOPC) modes, enabling nondestructive evaluation of its structural and electrical properties. The 4H-SiC polytype consists of Si–C bilayers stacked with a periodicity *n* = 4, forming a natural superlattice. The presence of folded phonon modes, such as the folded transverse optical (FTO) mode observed near 777 cm^−1^, confirms the *n* = 4 stacking sequence of the 4H-SiC structure. It is important to note that the Raman peaks shown in the spectra were observed in the regions of the laser-irradiated surface that were located away from the laser-irradiated track, specifically excluding the areas around the ridge lines where no peaks were detected. To assess the electrical properties of 4H-SiC samples, the LOPC modes at the *Γ* point that arise from the coupling between the LO phonon and free-carrier plasmon were analyzed. The plasmon frequency (*ω_ₚ_*), which represents the collective oscillation frequency of free electrons, is a key physical parameter for extracting the carrier concentration (*n*) through LOPC analysis. By examining the position and line shape of the LOPC features in the Raman spectrum and fitting them using a dielectric function model *ε*(*ω*) [58],

$$\varepsilon \left(\omega \right)=\varepsilon_{\infty }\left[1+\frac{\omega_{L}^{2}-\omega_{T}^{2}}{\omega_{T}^{2}-\omega^{2}-i\omega \Gamma }-\frac{\omega_{p}^{2}}{\omega \left(\omega +i\gamma \right)}\right]$$

the plasmon frequency *ω_ₚ_* was obtained.

The corresponding free-carrier concentration was then calculated using the standard plasmon relation.

$$\omega_{p}=\sqrt{\frac{4\pi n{\mathrm{e}}^{2}}{\varepsilon_{\infty }m}}$$

The analysis revealed that the 4H-SiC sample on the laser-irradiated surface possessed a carrier concentration (*n*) of (3.1 ± 0.1) × 10^18^ cm^−3^, demonstrating that Raman-based LOPC fitting provides an accurate and fully nondestructive method for evaluating the electrical properties of wide-bandgap semiconductors. In contrast, the laser-sliced surface exhibited two distinct carrier concentrations depending on the fitting model: approximately (3.5 ± 0.1) × 10^18^ cm^−3^ for Model 1 and nearly (2.0 ± 0.1) × 10^19^ cm^−3^ for Model 2. This variation likely reflects heterogeneous electronic responses associated with localized thermal histories, structural relaxation, or plasma-assisted material removal during slicing. Notably, SIMS analysis also revealed higher nitrogen concentrations on the laser-sliced surface, consistent with the observed differences in carrier concentrations and supporting the notion of electronic-structure and doping inhomogeneity. These results further confirm that 4H-SiC exhibits lower crystallographic anisotropy than 6H-SiC, leading to correspondingly weaker anisotropic behavior in its LOPC response.

Raman spectra were selectively collected only from locations within the mapping area where measurable Raman signals were detected. On rough surfaces, Raman peaks were not observed because the highly uneven topography—on the order of several micrometers, as confirmed by AFM—restricted proper optical focusing and significantly suppressed Raman signal acquisition. This behavior is clearly illustrated in Figure S4.

For the laser-irradiated surface, the intensity map revealed pronounced spatial contrast, particularly for the 776 cm^−1^ FTO mode, which showed periodic intensity enhancement aligned with the laser-scan direction. This pattern directly correlates with the surface morphology observed in both optical microscopy and AFM images, suggesting (i) localized modification of the stacking sequence, (ii) periodic strain accumulation along the irradiated scan path, and (iii) slight lattice relaxation or deformation induced by thermal gradients. The modes at 204 and 978 cm^−1^ also exhibited non-uniform spatial distributions, albeit with weaker contrast, indicating multiscale perturbations in the SiC lattice caused by localized heating and rapid cooling. Notably, the G-band at 1522 cm^−1^ appeared selectively in the irradiated regions, implying (i) laser-induced carbonization, (ii) defect-mediated Si–C bond breaking, and (iii) trace formation of graphitic residues near the surface. Such carbon-related features are commonly observed in high-fluence laser–SiC interactions and serve as reliable indicators of localized decomposition. In contrast, the non-irradiated regions exhibited distinctly different Raman characteristics. All four Raman modes displayed uniformly low intensities with negligible spatial variation across the mapping area. The 776 cm^−1^ FTO mode remained consistent with the response of pristine 4H-SiC, and the 1522 cm^−1^ G-band was nearly absent, confirming that carbonization and structural modification were strictly confined to the laser-exposed region. This uniformity clearly demonstrates that laser-induced perturbation is highly localized and does not propagate significantly beyond the irradiated track.

For the laser-sliced surface, SIMS measurements revealed a higher nitrogen concentration, which correlates with the observed variation in extracted carrier concentration and supports the presence of electronic inhomogeneity across the sliced surface. Figure S5 presents the Raman spectra and corresponding two-dimensional intensity maps obtained from both the laser-irradiated and laser-sliced surfaces, providing a comprehensive visualization and direct comparison of Raman features across the processed regions.

The electrical and Hall characteristics of the SiC wafer were evaluated using a four-point probe and the van der Pauw technique. The measurement configuration is shown in Figure S6, where the laser-sliced 4H-SiC wafer is mounted on a dedicated four-point-probe stage equipped with spring-loaded Au contacts and an edge connector for Hall and resistivity measurements. Both the front (laser-irradiated) and back (laser-sliced) surfaces were sequentially contacted to verify the electrical uniformity of the sample. The wafer was obtained by slicing a SiC ingot, followed by cleaning with acetone and drying to remove surface contaminants and residual stresses. Ohmic contacts were formed by deposition of Ni on the wafer surface followed by thermal annealing, ensuring stable electrical contact and minimizing errors arising from contact resistance. Hall and resistivity measurements were performed according to the standard van der Pauw formalism [58]. The extracted electron mobility was 88.3 cm^2^ V^−1^ s^−1^, which falls within the previously reported mobility range for heavily nitrogen-doped 4H-SiC (approximately 50–100 cm^2^ V^−1^ s^−1^). The bulk carrier concentration was determined to be −2.6 × 10^18^ cm^−3^, and the sheet carrier concentration was −3.2 × 10^16^ cm^−2^. The electrical conductivity and sheet resistance were measured as 38.0 Ω^−1^ cm^−1^ and 2.2 Ω^−1^, respectively. The average Hall coefficient was −2.3 cm^3^ C^−1^, corresponding to a resistivity of 2.6 × 10^−2^ Ω cm. The negative values of the Hall coefficient and carrier concentration confirm that electrons are the dominant carriers originating from the nitrogen donor doping. Importantly, the bulk carrier concentration obtained from the Hall measurements is in close agreement with the carrier density estimated from Raman LOPC analysis, which is consistent with earlier reports on phonon–plasmon interactions in SiC [59,60], indicating that both Hall and Raman methods provide reliable and mutually corroborating assessments of the electrical properties of the wafers.

### 3.4. Nanoscale Morphology and Phase Architecture

**Figure 5:**
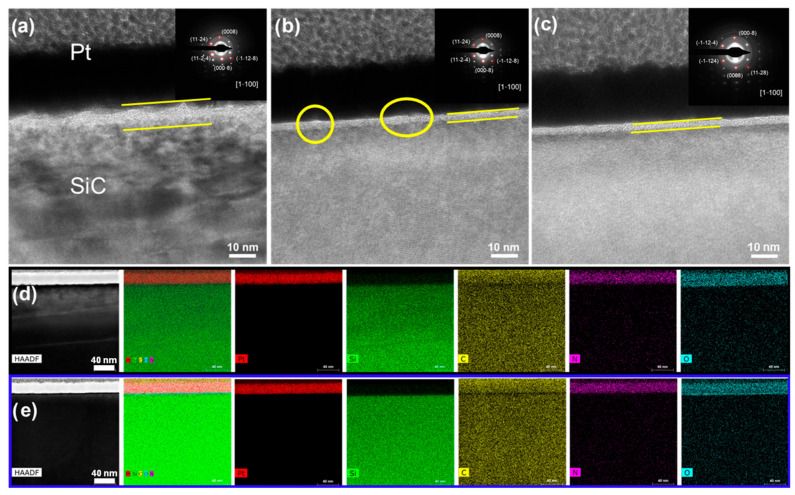
TEM, SAED, HAADF-STEM, and EDS analyses of laser-processed SiC. (**a**) Cross-sectional TEM image of the smooth laser-irradiated surface near the SiC/Pt interface. (**b**,**c**) Cross-sectional TEM images of the laser-sliced region, corresponding to the hill area (**b**) and valley area (**c**). The insets in (**a**–**c**) present the SAED patterns obtained from each region, confirming the crystalline orientation and structural features. (**d**,**e**) HAADF-STEM images and corresponding EDS elemental maps (Pt, Si, C, N, O) for the laser-irradiated side (**d**) and the laser-sliced side (**e**), illustrating the elemental distribution across the SiC bulk, interface, and laser-modified areas.

To analyze the damage (including phase transformation, lattice distortion, and microcracks) induced by laser irradiation in SiC at the atomic-level/nanoscale, FIB milling was used to prepare cross-sectional TEM specimens with a Pt capping layer as protection. Cross-sectional TEM images were acquired from laser-irradiated and laser-sliced sides. Figure 5a shows a cross-sectional TEM image of the SiC wafer near the laser-irradiated surface for the observation region located close to the SiC/Pt interface. Figure 5b presents a cross-sectional TEM image of the hill region on the laser-sliced surface, whereas Figure 5c shows the cross-section of the valley region of the laser-sliced surface. The corresponding SAED patterns are shown in the insets. A mixed layer consisting of amorphous and polycrystalline phases was observed between the Pt layer and bulk SiC on both the laser-irradiated and laser-sliced sides, as shown in the regions marked by yellow lines and circles in Figure 5a–c, with an average thickness of ~8 nm on the laser-irradiated side (Figure 5a) and ~2.8 nm and ~2.5 nm in the hill and valley regions of the laser-sliced surface, respectively (Figure 5b,c). The laser-irradiated side exhibited a thicker mixed-phase region, which was likely related to the additional Si–O species introduced by the chemical–mechanical polishing of the exposed surface. The bright spots and concentric rings with sharp boundaries in the SAED patterns confirmed the presence of a polycrystalline phase. The amorphous phase can be attributed to the presence of amorphous Si, C, or SiC. During laser irradiation, the instantaneous high temperature breaks the C–Si bonds in SiC; the lattice melts or even directly sublimates, and the molten or vaporized species are ejected and subsequently redeposited at the interface upon rapid cooling, forming an amorphous recast layer [5,43]. As shown in Figure 5a, several dark bands appear beneath the mixed-phase region. Importantly, continuous and nearly periodic lattice fringes were still visible within these bands. This contrast is more plausibly ascribed to thickness nonuniformity and curtaining artifacts introduced during FIB preparation or to localized carbon deposition under suboptimal vacuum conditions, rather than to any real phase or crystal structure transformation. A similar mixed-phase layer was observed on the laser-sliced side. However, the cross-section taken from the hill region (Figure 5b) shows a more corrugated SiC recast interface and a locally mottled contrast, whereas the valley region (Figure 5c) exhibits a much flatter and smoother interface with a more uniform contrast. This indicates that the structural damage and recast-layer roughening were more severe on the hills than on the valleys, which is consistent with the AFM results presented in Figure 3 where the hill region shows significantly higher R_a_ and R_rms_ values than the valley region. All SAED patterns in Figure 5a–c show sharp well-ordered diffraction spots that can be consistently indexed to the [1–100] zone axis of 4H-SiC, corresponding to the P63mc space group (a = b = 3.08 Å, c = 10.07 Å). The diffraction patterns from the laser-irradiated region and from both the hill and valley regions of the laser-sliced surface are essentially identical, forming a uniform [1–100] spot array. This indicates that the macroscopic crystal orientation of the wafer remains intact after laser processing and that the damage is confined to a very thin near-surface region. The bulk crystallinity of the laser-processed SiC was further examined using FIB-TEM, as shown in Figures S7 and S8. The SEM images presented in Figure S7a,d show the FIB-prepared lamellae removed from the laser-irradiated surface and the hill region of the laser-sliced surface, respectively. Cross-sectional HRTEM images of the bulk regions of these lamellae (Figure S8) revealed well-ordered atomic planes with no observable lattice distortion or amorphous layers. The insets in Figure S8 show the corresponding SAED patterns indexed along the [1–100] zone axis, displaying sharp and regularly spaced diffraction spots characteristic of single-crystalline 4H-SiC and confirming identical crystal orientations on both sides. No additional reflections or streaks associated with polytype transformations, secondary phases, or defect clustering were observed, indicating that the bulk region remained structurally intact after laser irradiation and internal slicing. Figure 5d,e shows HAADF-STEM images and EDS mapping results for the laser-irradiated and laser-sliced sides, respectively. Si and C were uniformly distributed in both regions, consistent with the SiC matrix, whereas Pt was confined to the top protective layer with no detectable diffusion into the substrate. A continuous O-rich band was observed immediately beneath the Pt layer on the laser-irradiated side; a similar O-enriched band was also present on the laser-sliced side but was significantly thinner. The N signal was weak and nearly uniform, which is consistent with light homogeneous N doping. To complement the qualitative maps, STEM–EDS spectra were collected at several positions across the Pt/SiC cross-section on both sides of the wafer (Figure S9 in the Supporting Information). In each case, the spectrum acquired from the near-surface region just beneath the Pt cap (first ROI) exhibits a clearly stronger O K_α_ peak and slightly enhanced C intensity relative to the spectra from deeper regions, whereas the spectrum taken from the bulk (third ROI) is dominated by Si and C with only a very weak O signal close to the background level. These trends indicate that oxygen (and a small amount of additional carbon) is concentrated in a thin near-surface band, while the underlying bulk is dominated by Si and C signals with only a minor oxygen contribution. However, due to the limited sensitivity and depth resolution of EDS for light elements in thin lamellae, these results are treated in a semi-quantitative manner and are combined with ARXPS and SIMS to more accurately establish the depth profile of oxygen within the laser-modified region.

### 3.5. Surface Chemistry and Bonding Characteristic

**Figure 6:**
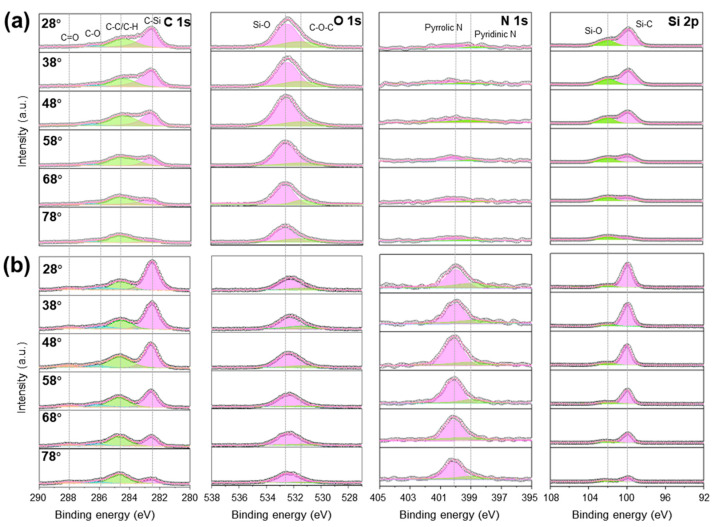
Angle-resolved XPS spectra of laser-processed SiC surfaces. (**a**) ARXPS spectra of the laser-irradiated surface and (**b**) the laser-sliced surface, showing the C 1s, O 1s, N 1s, and Si 2p core-level regions measured at take-off angles ranging from 28° to 78°. All spectra were charge-corrected by referencing the adventitious C 1s (C–C/C–H) peak at 284.6 eV. The surface-normal direction was defined as 0°, with high take-off angles corresponding to surface-parallel emission. During ARXPS acquisition, the X-ray incidence angle remained fixed, and photoelectrons were collected simultaneously along six emission directions using a hemispherical analyzer.

ARXPS measurements were performed to investigate the surface chemical composition changes induced by laser irradiation and to conduct a detailed depth analysis. Figure 6 illustrates the high-resolution XPS spectra obtained for the (a) laser-irradiated and (b) laser-sliced surfaces. The spectra cover key chemical bonding states, including C 1s, O 1s, N 1s, and Si 2p, measured at take-off angles between 28° and 78° with an angle of 0° defined as perpendicular to the sample surface. The full spectrum obtained from the ARXPS analysis is shown in Figure S10, and the detailed fitting parameters (peak positions, FWHM, and molar fractions of each chemical state) are summarized in Tables S3 and S4 for the laser-irradiated and laser-sliced surfaces, providing quantitative insights into the bonding environments and surface chemical modifications induced by laser irradiation. The atomic percentage variations in these elements as functions of the takeoff angle for the two surfaces are presented in Figure S10c,d. It is important to note that a larger sampling angle probes shallower regions near the sample surface, providing more information about the surface chemical composition.

In the C 1s spectrum, four components were resolved at 282.6, 284.6, 286.0, and 288.0 eV, which are assigned to C–Si, C–C, C–O/C–H, and C=O, respectively. The C–Si peak reflects the intrinsic covalent bonding in SiC. The C–C peak arises from an adventitious carbon overlayer and may also contain contributions from the laser-induced carbonaceous species (amorphous/graphitic/diamond-like carbon) formed by Si–C bond scission and rapid resolidification on the laser-sliced surface. C–O and C=O originate from the oxygenated carbon formed at the surface during laser processing and subsequent air exposure. The C–H signal is generally attributed to adventitious hydrocarbons on the surface, with minor contributions from hydrocarbons generated during the laser slicing process or reactions during the cleaning process in air. In ARXPS where the take-off angle is referenced to the surface normal, increasing the angle makes the analysis more surface-sensitive; accordingly, on the laser-sliced side, the relative intensity of C–C increases at larger angles, whereas at smaller angles, the intensity of C–Si increases. For the laser-irradiated surface, the C–O/C=O fractions decreased markedly compared with the laser-sliced side, and the same result was obtained for each angle. This can be attributed to the mechanical fracture process experienced by the laser-cut surface which exposed more C dangling bonds. These dangling carbon bonds are highly reactive and readily react with oxygen atoms from ambient water vapor or atmospheric oxygen, leading to the formation of higher concentrations of C–O and C=O bonds. In O 1s, two peaks attributed to Si–O and C–O–C were observed at 532.5 and 531.5 eV, respectively. For a laser-sliced surface, the Si–O bonds originate from silicon oxide generated by the reaction of SiC with oxygen in air during laser cutting [8]. The proportion of Si–O bonds was higher on the laser-irradiated surface than on the laser-sliced surface. This is primarily because the laser-irradiated surface formed abundant Si–O bonds during the laser separation process; as evidenced by the SEM/EDS results discussed above, additional Si–O bonds were generated during the polishing process, further enriching the Si–O content on this surface. The increase in the O 1s and Si 2p–SiO fractions at higher angles shown in Figure S10c,d indicates the presence of a thin surface oxide layer, whereas the decreasing Si 2p–SiC contribution reflects reduced sampling of the underlying SiC lattice at surface-sensitive geometries. These trends collectively confirm that both surfaces exhibit a modified near-surface region confined to the top few nanometers, with the chemical bonding of bulk SiC remaining intact. The C–O–C peak may originate from the surface residual carbon (C–C) generated by laser cutting and the oxygen-containing functional groups formed by the reaction of the terminal carbon of the broken C–Si bond with oxygen. In N 1s, two peaks at 398.9 eV (pyridinic N) and 400.0 eV (pyrrolic N) can be detected, particularly on the laser-sliced side, consistent with nitrogen activation/incorporation into a carbon-rich surface layer at elevated temperatures during laser cutting processing (and subsequent adsorption). By contrast, the laser-irradiated surface exhibited almost no detectable nitrogen signal owing to the removal of dangling carbon bonds and surface microcracks during chemical–mechanical polishing, eliminating most chemically active sites for nitrogen incorporation. Unlike oxygen, which can form stable Si–O bonds directly with exposed Si atoms regardless of the availability of dangling carbon bonds, nitrogen incorporation depends strongly on the presence of reactive dangling carbon bond sites. Therefore, on a laser-irradiated surface where dangling carbon bonds are largely absent, nitrogen adsorption is severely limited. This indicates that nitrogen incorporation is dangling-carbon-bond-dependent, in contrast to oxygen oxidation, which is Si-dependent. In the Si 2p spectrum, the component at 102 eV is assigned to Si–O, reflecting the silicon oxide formed by the reaction of SiC with oxygen, whereas the peak at 100 eV corresponds to Si–C, representing the SiC lattice. As expected, the relative contribution of Si–C increased with greater depth, which is consistent with the sampling of more bulk SiC. Figure S11 summarizes the relative atomic percentages of the deconvoluted C 1s, O 1s, N 1s, and Si 2p components for both surfaces as a function of the take-off angle, which provides depth-sensitive information about the carbon bonding states, oxygen-containing surface groups, nitrogen configurations, and silicon oxidation states, enabling comparative evaluation of the near-surface chemical structures of the laser-irradiated and laser-sliced SiC surfaces. On the laser-irradiated surface, the Si–O fraction was the highest at large take-off angles and gradually decreased toward smaller angles, whereas the Si–C fraction exhibited the opposite trend. This behavior is consistent with the O 1s analysis and confirms the presence of a thin SiOₓ-rich layer confined to the near-surface region, beneath which the bulk chemistry remains dominated by Si–C bonding.

**Figure 7:**
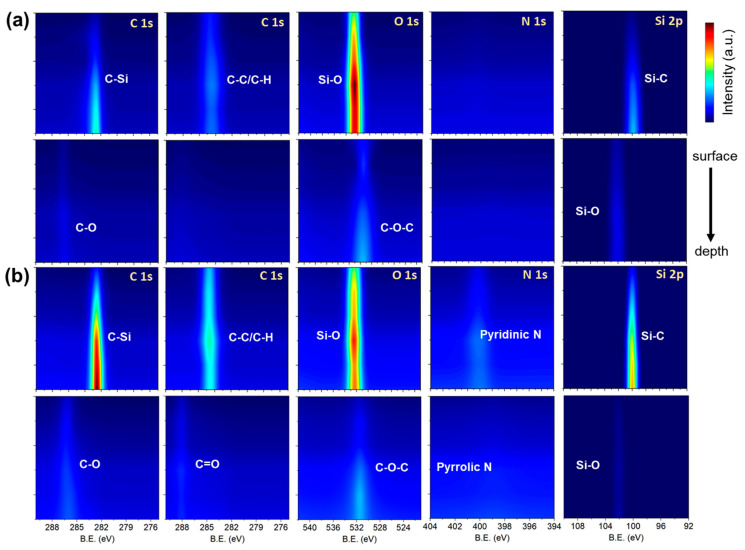
Two-dimensional XPS chemical-state maps of laser-processed SiC surfaces. (**a**,**b**) Chemical-state mapping of the laser-irradiated surface (**a**) and the laser-sliced surface (**b**) obtained from deconvoluted C 1s, O 1s, N 1s, and Si 2p spectra. The mapped components include C–Si (~283.0 eV), C–C/C–H (~285.0 eV), C–O/C=O (~286–288 eV), Si–C (~100.5 eV), and Si–O (~103.5 eV). The laser-sliced surface additionally shows nitrogen-related signals assigned to pyridinic N (~398.4 eV) and pyrrolic N (~400.3 eV). The color contrast reflects the relative intensities of each bonding state, enabling comparison between the spatial chemical distributions of the two surfaces.

Figure 7 presents two-dimensional XPS mapping results for the laser-irradiated and laser-sliced SiC surfaces, highlighting the evolution of the chemical bonding states induced by laser irradiation. On the laser-irradiated surface (upper panels), the C 1s spectra were dominated by the C–Si component at 283.0 eV, with minor contributions from the surface hydrocarbons (C–C/C–H) near 285.0 eV. The O 1s signal shows a weak Si–O contribution, which is consistent with the thin native oxide layer formed upon exposure to air. The Si 2p spectra reveal a sharp Si–C doublet at 100.3–100.7 eV, confirming the well-ordered SiC lattice with minimal surface oxidation. No significant N 1s peak was detected, indicating negligible nitrogen incorporation into the as-grown crystals. By contrast, the laser-sliced surface (lower panels) exhibits a clear chemical modification. The C 1s region still contains C–Si and C–C/C–H peaks but shows increased intensity in the C–O (286.3 eV) and C=O (287.8 eV) components, suggesting surface oxidation and partial carbon rehybridization due to high local temperature and photochemical effects. The O 1s spectra exhibit stronger Si–O (∼532.5 eV) and C–O–C signals, indicating the formation of suboxide species at the Si–C interface. Notably, the N 1s spectra reveal two distinct peaks at approximately 398.4 eV (pyridinic N) and 400.3 eV (pyrrolic N), attributed to nitrogen incorporation during laser irradiation in N_2_-purged atmosphere. This suggests that the UV laser interaction locally activates nitrogen radicals, enabling bonding with defect sites or graphitic carbon fragments on the surface. The Si 2p spectra maintained the main Si–C component but with a broadened tail toward higher binding energies, confirming partial oxidation and amorphization near the slicing interface. Overall, the XPS mapping data demonstrate that the UV laser slicing process not only induces mechanical separation, but also leads to chemical modification of the SiC surface, including oxidation and nitrogen incorporation. These surface states are expected to influence the electrical contact behavior and interfacial bonding in the subsequent device fabrication and metallization steps.

### 3.6. Elemental Distribution and Interface Evolution

**Figure 8:**
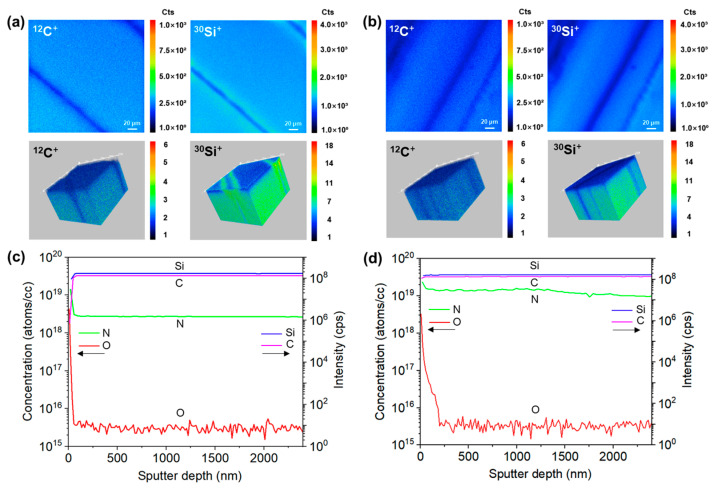
SIMS images and depth profiles of laser-processed SiC surfaces. (**a**,**b**) O_2_^+^-beam SIMS ^12^C^+^ and ^30^Si^+^ ion images (2D and 3D) from the laser-irradiated surface (**a**) and the laser-sliced surface (**b**) over a 250 × 250 μm^2^ area. (**c**,**d**) Cs^+^-beam SIMS depth profiles for the laser-irradiated (**c**) and laser-sliced (**d**) surfaces, showing N and O concentrations (left axis) and Si and C matrix ion intensities (right axis) as a function of sputter depth. The arrows in panels (**c**,**d**) indicate the corresponding y-axes: the left-pointing arrow denotes the concentration axis (atoms/cc), whereas the right-pointing arrow denotes the intensity axis (cps).

Figure 8 shows the SIMS imaging and depth analysis results for the laser-irradiated and laser-sliced surfaces. In the O_2_^+^ beam 2D and 3D imaging (Figure 8a,b), the ^12^C^+^ and ^30^Si^+^ ion signals from both the laser-irradiated and laser-sliced sides exhibit diagonal streaks along the cutting direction, corresponding to the laser cutting track and the surrounding recast layers. On the laser-irradiated surface, the ^12^C^+^ signal near the recast layer diminishes and the ^30^Si^+^ signal also weakens. However, a region with enhanced ^30^Si^+^ signal appears around the cutting track, which is attributed to the formation of a Si-rich silica layer in the recast region. During the laser cutting process, the surface region was strongly heated and partially melted in ambient air. Under these conditions, SiC decomposes and carbon preferentially evaporates as C, CO and CO_2_, leaving a Si-enriched melt. The residual Si subsequently reacts with oxygen to form a Si-enriched oxide layer along the edge of the recast zone. On the laser-irradiated surface, except for the region around the laser cutting track where the ^30^Si^+^ signal is significantly stronger, the ^30^Si^+^ signal in other areas is weaker compared to the bulk. This is due to the surface Si evaporation caused by laser heating and the formation of a silica layer on the surface which dilute the surface Si concentration. Additionally, the evaporation of carbon leads to a lower intensity of the ^12^C^+^ signal at the surface compared to the bulk. On the laser-sliced surface, the ^12^C^+^ signal shows little difference between the surface and bulk because the sliced surface originates from an internal delamination plane which is largely shielded from oxygen during laser processing, making it difficult for carbon atoms to oxidize and evaporate. By contrast, the ^30^Si^+^ signal at the surface is lower than that in the bulk due to the formation of a thin SiO_2_ layer on the surface which reduces the surface Si concentration. In the Cs^+^ beam depth analysis (Figure 8c,d), the secondary ion signals of Si and C maintain a plateau after entering the bulk, indicating a homogeneous composition of the substrate. Using the density and molar mass of 4H-SiC, the total atomic density of the Si + C matrix was estimated to be 9.64 × 10^22^ atoms·cm^−3^. On the laser-irradiated side (bulk region), the N concentration ranges from 2.65 × 10^18^ to 3.06 × 10^18^ atoms·cm^−3^, corresponding to an atomic percentage of approximately 2.75–3.17 × 10^−3^%. The O concentration ranges from 2.45 × 10^15^ to 3.95 × 10^15^ atoms·cm^−3^, giving an atomic percentage of approximately 2.54–4.10 × 10^−6^%. On the laser-sliced side, the N concentration lies between 9.61 × 10^18^ and 1.58 × 10^19^ atoms·cm^−3^, corresponding to an atomic percentage of roughly 1.00–1.64 × 10^−2^% while the O concentration is between 3.8 × 10^15^ and 9.79 × 10^16^ atoms·cm^−3^, corresponding to atomic percentage of 3.94 × 10^−6^–1.02 × 10^−4^%. Therefore, even in the near-surface region where oxygen is enriched, the combined fraction of N and O atoms remains below ~0.03%, indicating that the SiC matrix is overwhelmingly composed of Si and C, and that laser processing mainly redistributes these dilute impurities without significantly changing the overall stoichiometry. On the laser-irradiated side, a prominent oxygen peak appeared near a sputtering depth of 0 nm and decreased rapidly, suggesting a significantly oxygen-enriched layer in the top few tens of nanometers. By contrast, the oxygen peak on the laser-sliced surface was noticeably lower; however, the oxygen concentration decreased more slowly with depth compared to that on the laser-irradiated surface. This can be attributed to the sidewall effects. During the measurement, the Cs^+^ ion beam is incident on the surface at an angle of 23.5° relative to the surface, and secondary ions are detected at an angle of 90°. Owing to the surface roughness of the laser-sliced area, at different sputter depths the detector captures not only oxygen ion signals originating from the surface but also those from the sidewall regions that are relatively less sputtered where oxygen remains owing to incomplete ion removal. This results in a slower decrease in the oxygen concentration with greater depth.

Figure S12 presents optical microscopy images of the ion-etched craters generated during the SIMS depth-profiling experiments on the two SiC surfaces. Figure S12a shows a crater formed on the laser-irradiated surface, where the sputtered area (marked by the red box) is relatively flat and featureless. Figure S12b shows the corresponding crater on the laser-sliced surface, which contains a pronounced hill–valley relief and wave-like undulations produced during the slicing process. The area where the ion beam was rastered to produce the crater for analysis is marked by the red box. These morphology differences lead to distinct sputtering responses: on the laser-sliced side, the inclined sidewalls inside the crater give rise to a side-wall effect so that oxygen-rich hill regions continue to contribute to the secondary-ion signal as the crater deepens, consistent with the more gradual decay of the O profile in the SIMS depth curves. The nitrogen concentration on both the laser-irradiated and laser-sliced surfaces was higher at the surface and decreased with greater depth. This was likely due to the adsorption of nitrogen from the environment during the laser slicing process. However, the nitrogen concentration on the laser-sliced surface was initially higher than that on the laser-irradiated surface which can be attributed to the higher oxide layer on the laser-irradiated surface masking the nitrogen signal. Moreover, as shown by the XPS analysis, during the CMP process, the surface temperature increased and chemical corrosion led to the evaporation of nitrogen from the laser-irradiated surface. Additionally, the XPS data indicated that after polishing, the laser-irradiated surface had fewer active sites available for nitrogen adsorption, further reducing the observed surface nitrogen signal. By contrast, the nitrogen concentration in the bulk of the laser-sliced surface was an order of magnitude higher than that of the laser-irradiated surface, mainly because of the volatilization and oxidation of nitrogen at high temperatures, resulting in a lower nitrogen concentration in the bulk. As the sputtering depth increases, the Si and C signal intensities on both the laser-irradiated and laser-sliced surfaces initially increase and then gradually stabilize. This phenomenon is primarily influenced by the surface oxide layer. During the initial sputtering stage, the surface oxide layer decreases the Si and C signals. As sputtering progressed, the oxide layer was gradually removed, exposing the unoxidized Si and C and leading to a gradual increase in the signal intensity, which eventually stabilized.

### 3.7. Surface Features of UV Laser-Sliced SiC Wafers

**Figure 9:**
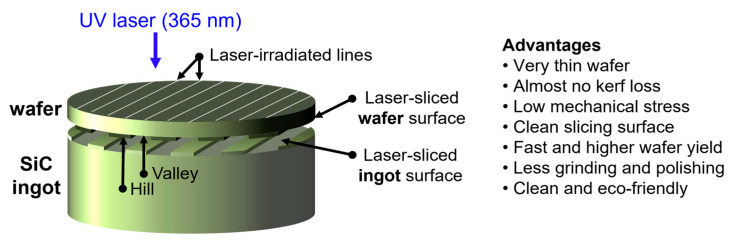
Schematic illustration of the UV laser slicing process and the resulting separation surface morphology. A focused UV laser (λ = 365 nm) is scanned across the SiC ingot to create a periodic subsurface modified layer that serves as the cleavage plane. The detached wafer exhibits a well-defined hill–valley structure that directly reflects the laser scanning pitch, with the hill region showing slightly higher roughness due to localized laser focusing. This laser-based slicing method provides key advantages, including near-zero kerf loss, clean and low-damage separation surfaces, high material utilization, and efficient production of ultra-thin SiC wafers.

The hill–valley structure generated by laser slicing was determined by the laser scanning conditions (Figure 9). Analysis results indicate that the width of the hill regions is approximately 70–80 μm, which is due to the effect of the localized laser focusing. The width of the valley regions can be controlled by adjusting the laser scanning interval, and was found to be approximately 200 μm in this study. Because the current settings are not optimal, the widths of the hill and valley regions can be partially adjusted. Narrower valley regions facilitate wafer separation after laser slicing; however, to improve the surface roughness, it is necessary to broaden the valley width and adjust the laser parameters, such as the scanning interval and pulse width. Additional studies are currently underway to determine the optimal conditions for 8-inch wafers, as well as to reduce the wafer thickness from its current value of 120 μm. Such optimization can minimize the surface roughness and localized damage during the laser slicing process, thereby simplifying subsequent processing and enhancing the overall wafer quality.

## 4. Conclusions

In this study, the effects of UV laser slicing on heavily N-doped 4° off-axis 4H-SiC wafers were systematically investigated. The results confirmed that laser slicing preserved the 4H-SiC crystal structure while leaving the [0001] crystallographic direction and macroscopic crystal orientation intact, as evidenced by the X-ray diffraction and pole-figure measurements. Raman spectroscopy, including analysis of the FTO mode and LOPC modes, provided a free-carrier concentration of approximately 3.1 × 10^18^ cm^−3^, consistent with the results obtained from Hall measurements. These findings suggest that the electrical properties of the laser-sliced 4H-SiC are comparable to those of power-device-grade materials. Multi-scale analyses using AFM, XPS, SIMS, and TEM/SAED revealed notable nanoscale surface modifications, particularly in the laser-sliced regions. A thin, mixed amorphous/polycrystalline layer and an oxygen-rich region were observed near the surface, with increased roughness and thicker modified layers present in the hilly regions of the laser-sliced surface compared with the smoother laser-irradiated side and valley regions. The laser-irradiated surface showed higher oxygen enrichment, likely due to the prior chemical–mechanical polishing. These findings indicate that although laser slicing does not affect the intrinsic crystal structure of 4H-SiC, it introduces localized surface damage, particularly in the form of surface roughness and oxidation, which is confined to the top tens of nanometers. This surface modification must be controlled through subsequent surface-finishing steps to ensure the overall quality of the laser-sliced 4H-SiC wafer for device applications. In future work, the present experimental characterization will be complemented by multi-scale numerical modeling, such as combining continuum or molecular-dynamics simulations with ultrafast energy-deposition models, to establish quantitative links between laser slicing parameters, defect formation, and the resulting surface and interface properties.

## Data Availability

The original contributions presented in this study are included in the article/Supplementary Material. Further inquiries can be directed to the corresponding author.

## Supplementary Materials

The following supporting information can be downloaded at: https://www.mdpi.com/article/10.3390/ma18245615/s1, Figure S1: Schematic of the UV picosecond laser slicing setup. Figure S2: Photographs of the 8-inch wafer after laser slicing from N-doped 4H-SiC single crystal ingot. Figure S3: AFM images and corresponding line-profile plots obtained from various regions of the 4H-SiC wafer. Figure S4: Raman spectra from rough parts. Figure S5: Raman spectra and corresponding two-dimensional intensity maps of characteristic vibrational modes obtained from the laser-irradiated surface of the 4H-SiC wafer. Figure S6: Photograph of the 4H-SiC sample mounted on a four-point probe stage for electrical measurements. Figure S7: SEM images of FIB-prepared lamellae from the SiC surface and bulk regions. Figure S8: TEM characterization of the two SiC surface. Figure S9: STEM–EDS maps and spectra for the 4H-SiC wafer. Figure S10: ARXPS survey spectra obtained from the two SiC surface and the angular dependence of the atomic percentages for Si2p–SiC, Si2p–SiO, C 1s, N 1s, and O 1s extracted from the two SiC surface. Figure S11: Relative atomic percentages of the deconvoluted chemical components obtained from the two SiC surface at take-off angles ranging from 28° to 78°. Figure S12: Optical microscope images of the ion-etched craters formed during the SIMS depth profiling measurements on the two SiC surface. Table S1: The key laser processing conditions employed in the present study and compares them with representative parameters from earlier laser-processing reports on SiC. Table S2: The elemental compositions obtained from EDS measurements performed on the two SiC surfaces. Table S3: The detailed fitting results of the deconvoluted XPS peaks obtained from the laser-irradiated SiC surface. Table S4: The detailed fitting results of the deconvoluted XPS peaks obtained from the laser-sliced SiC surface. References [6,8,13,17,27,61,62,63,64,65,66,67] are cited in the Supplementary Materials.

## Abbreviations

The following abbreviations are used in this manuscript:

SiC	Silicon Carbide
XPS	X-ray Photoelectron Spectroscopy
SIMS	Secondary Ion Mass Spectrometry
FIB	Focused Ion Beam
TEM	Transmission Electron Microscopy
SAED	Selected Area Electron Diffraction
AFM	Atomic Force Microscopy
ARXPS	Angle-Resolved X-Ray Photoelectron Spectroscopy
FTO	Folded Transverse Optical
LOPC	Longitudinal Optical-Phonon Coupled
FWHM	Full Width at Half Maximum
EV	Electric Vehicle
CMP	Chemical Mechanical Planarization
UV	Ultraviolet
