# Supplementary material for: Comprehensive Structural and Interfacial Characterization of Laser-Sliced SiC Wafers
Source: Materials (Basel). 2025 Dec 14;18(24):5615. doi: 10.3390/ma18245615 (PMC12734574; doi:10.3390/ma18245615)
Supplement: Supplementary file 1 [file materials-18-05615-s001.zip › materials-4027382-supplementary.pdf]

Supplementary Materials

Comprehensive Structural and Interfacial Characterization of Laser-Sliced SiC Wafers

Hong Chen[1,†], Seul Lee[1,†], Minseung Kang[1], Hye Seon Youn[1], Seongwon Go[1], Eunsook Kang[3], and Chae-Ryong Cho[1,2*]

[1] Department of Nano Fusion Technology, Pusan National University, Busan 46241, Republic of Korea
[2] Department of Nano Energy Engineering, Pusan National University, Busan 46241, Republic of Korea
[3] R&D center, Unis Co., Ltd. 1394 Nakdong-daero, Sasang-gu, Busan 46907, Republic of Korea
* Correspondence: crcho@pusan.ac.kr (C.-R. C.); Tel.: +82-51-510-6114 (C.-R.C.)
† These authors contributed equally to this study.

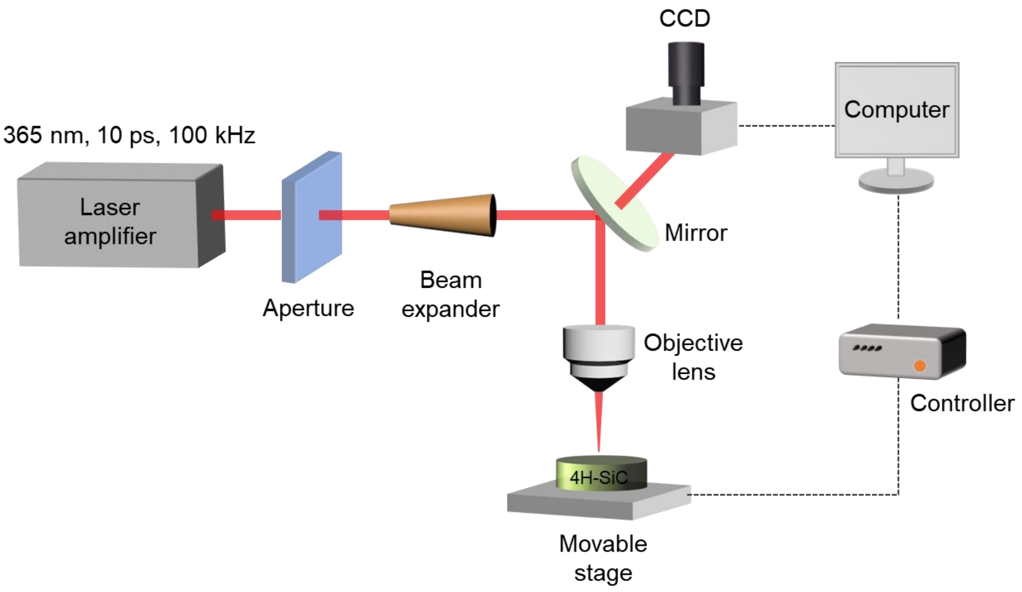

Figure S1. Schematic of the UV picosecond laser slicing setup.

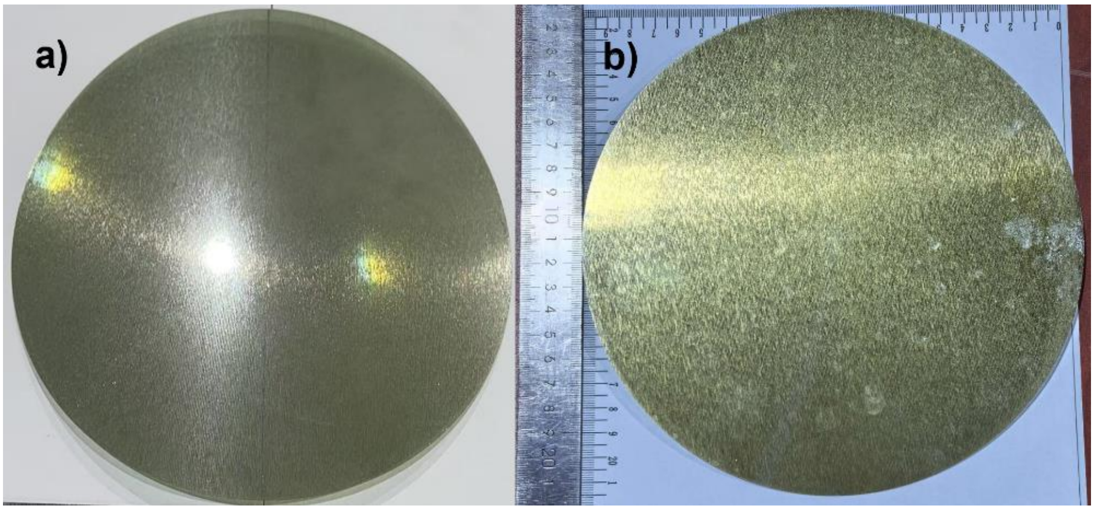

Figure S2. Photographs of 8-inch wafer after laser slicing from N-doped 4H-SiC single crystal ingot. Photographs of 8-inch wafer after laser slicing from N-doped 4H-SiC single crystal ingot. (a) Laser-irradiated side, showing periodic surface texturing generated by surface laser scanning prior to wafer separation. (b) Laser-sliced side corresponding to the internal fracture plane formed at the laser focal depth.

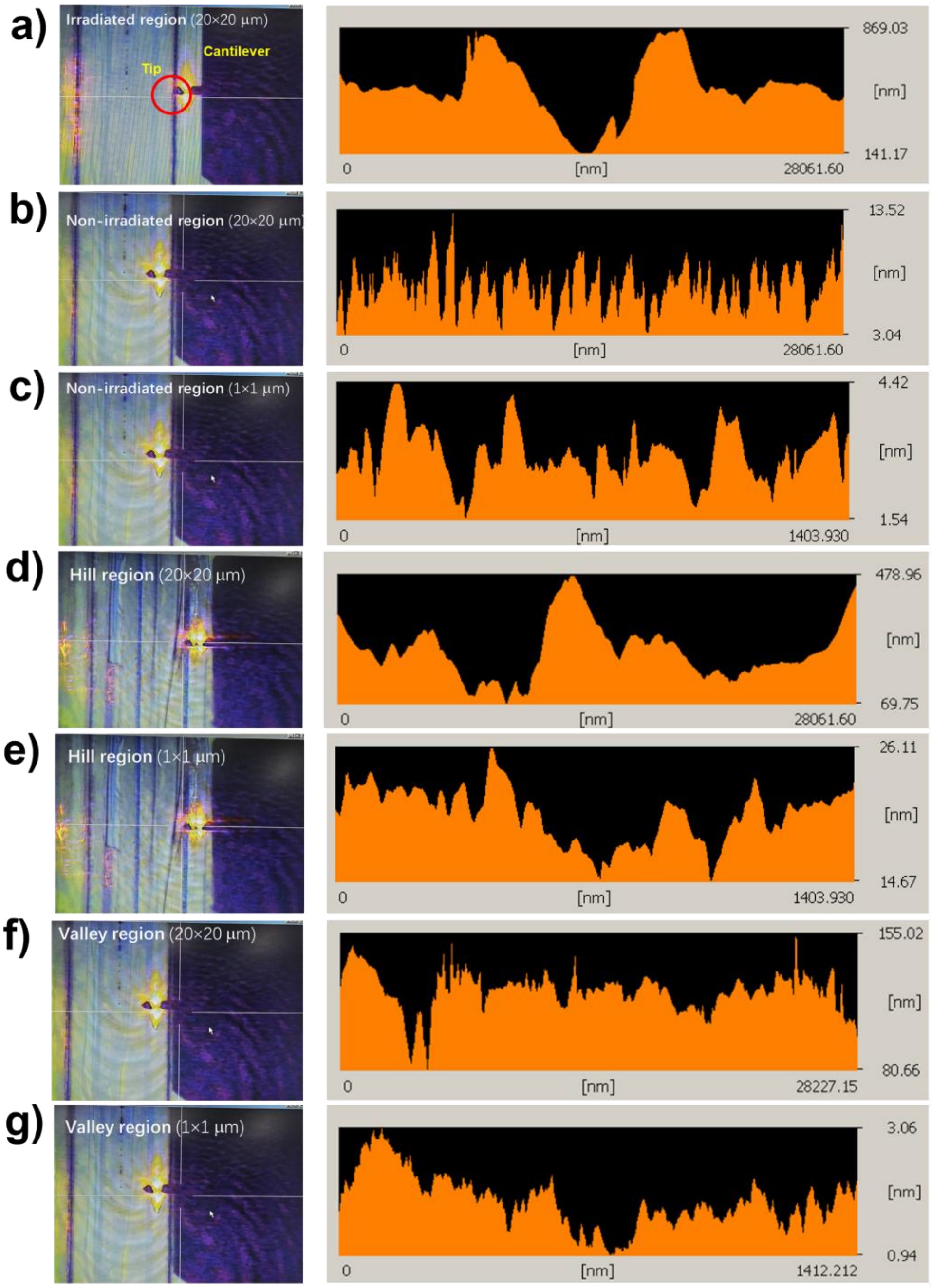

Figure S3. AFM images and corresponding line-profile plots obtained from various regions of the 4H-SiC wafer. For each panel, the **left** image shows the AFM topography with the marked scan trace, and the **right** plot shows the corresponding height profile along that trace. **(a)** Laser-irradiated region (20 × 20 µm²). **(b)** Non-irradiated region on the laser-irradiated side (20 × 20 µm²). **(c)** Non-irradiated region on the laser-irradiated side (1 × 1 µm²). **(d)** Hill region on the laser-sliced side (20 × 20 µm²). **(e)** Hill region on the laser-sliced side (1 × 1 µm²). **(f)** Valley region on the laser-sliced side (20 × 20 µm²). **(g)** Valley region on the laser-sliced side (1 × 1 µm²).

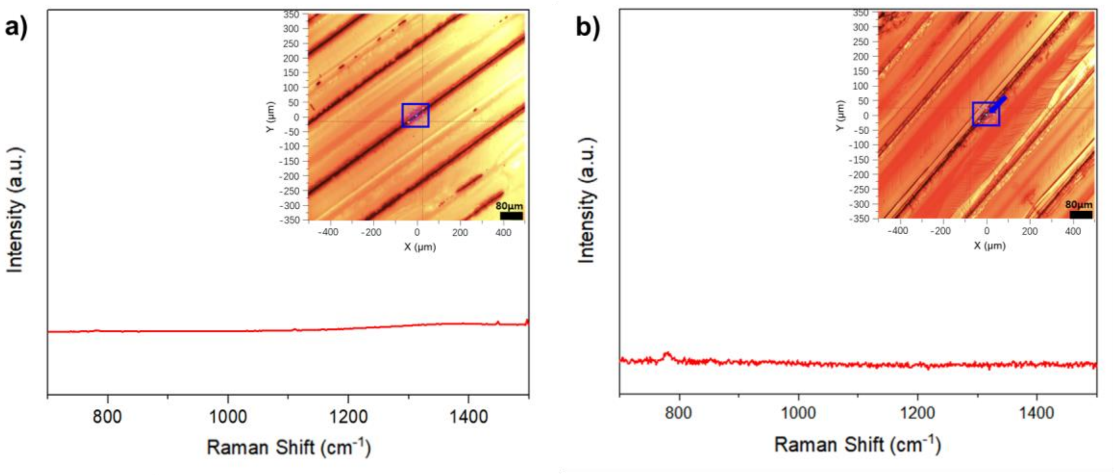

Figure S4. Raman spectra from rough parts. (a) laser-irradiated and (b) laser-sliced surfaces. In both surfaces, no distinct Raman peaks are observed in the rough regions.

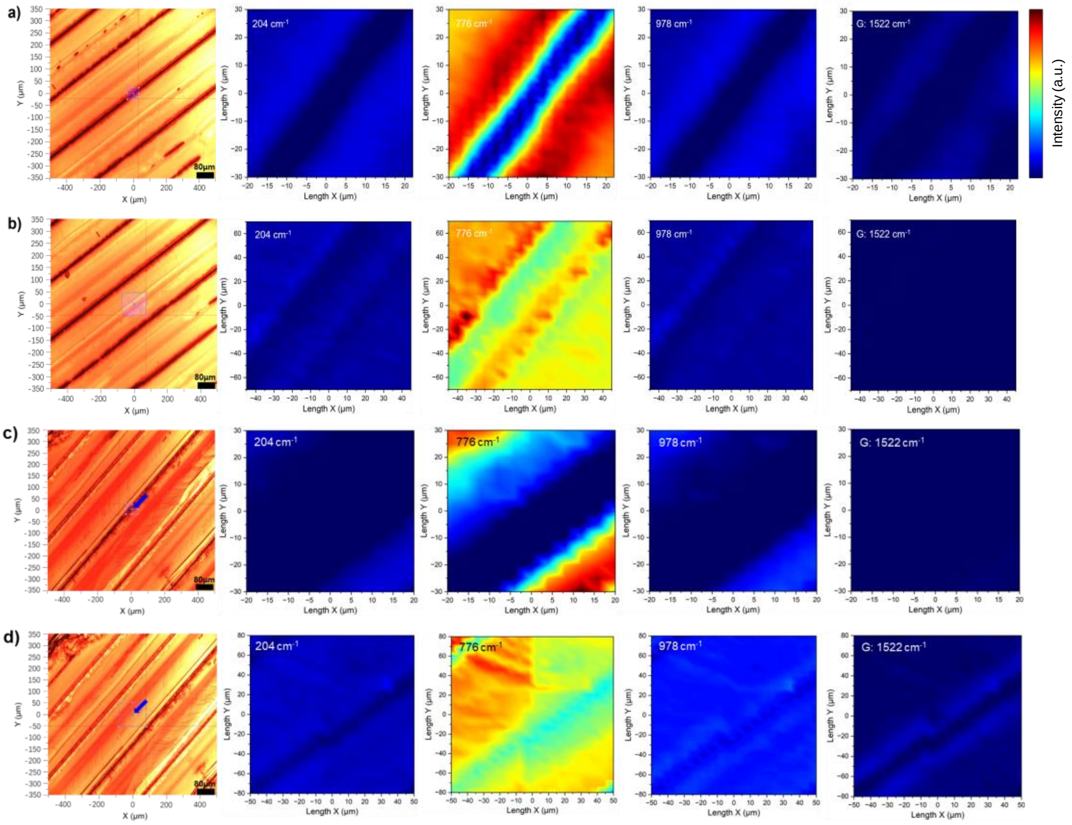

Figure S5. Raman spectra and corresponding two-dimensional intensity maps of characteristic vibrational modes obtained from the laser-irradiated surface of the 4H-SiC wafer. Raman spectra and corresponding two-dimensional intensity maps of characteristic vibrational modes obtained from the laser-irradiated surface of the 4H-SiC wafer. (a) Laser-irradiated region, (b) non-irradiated region on the same surface, (c) Hill region and (d) Valley region on the same surface. For each row, the first panel shows the optical micrograph of the mapped area, while the subsequent panels (from left to right) display the integrated-intensity maps of the Raman peaks at 204, 776, 978, and 1522 cm^{-1} (G band). These Raman modes correspond to low-frequency lattice vibrations (204 cm^{-1}), the folded transverse optical phonon mode characteristic of 4H-SiC (776 cm^{-1}), and higher-frequency phonon modes (978 cm^{-1}), while the 1522 cm^{-1} band reflects carbon-related signatures. The contrast differences in the intensity maps clearly reveal how both the laser-irradiated versus non-irradiated regions and the hill versus valley areas exhibit distinct modifications in the local lattice structure and phonon response.

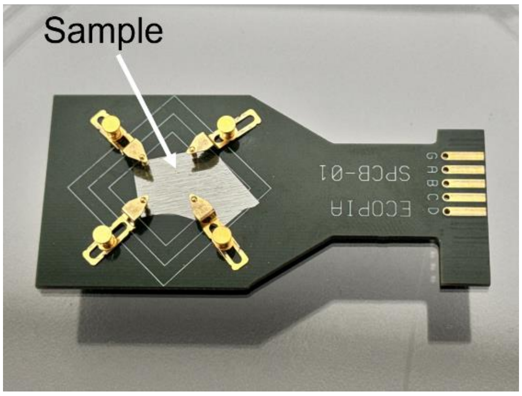

Figure S6. Photograph of the 4H-SiC sample mounted on a four-point probe stage for electrical measurements. The 4H-SiC specimen was secured on a dedicated measurement platform equipped with spring-loaded gold contacts for four-point probe, Hall effect, and resistivity measurements. Both the front and back surfaces of the wafer were sequentially characterized to evaluate carrier transport properties and verify the electrical uniformity of the laser-processed sample.

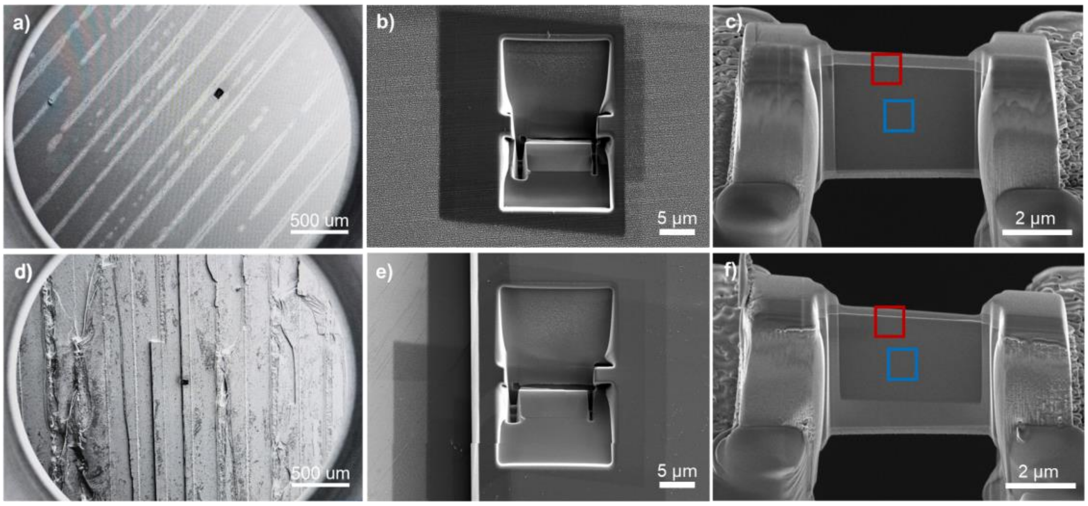

Figure S7. SEM images of FIB-prepared lamellae from the SiC surface and bulk regions. (a,d) SEM images showing the FIB-prepared lamellae extracted from the laser-irradiated surface and the laser-sliced surface, respectively. (b,e) Enlarged views of the regions marked in black in (a) and (d) after FIB processing. (c,f) Final FIB-prepared samples, where the red-marked areas were used for EDS measurements and the blue-marked areas were used for TEM characterization.

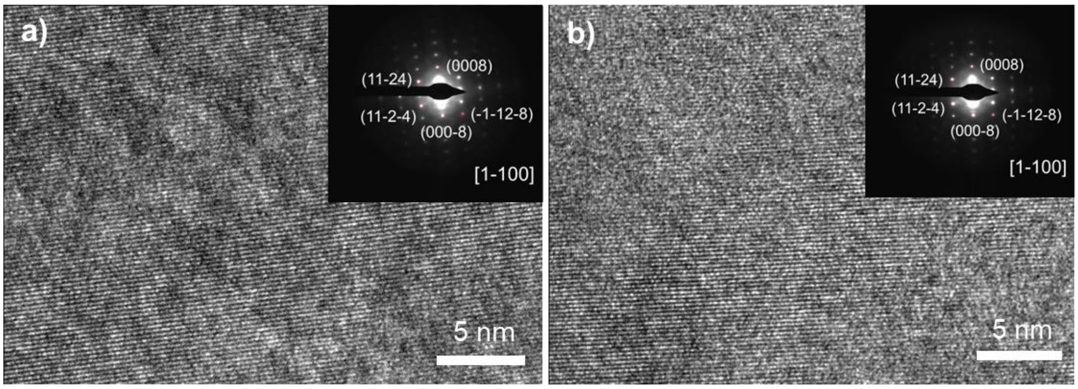

Figure S8. TEM characterization of the two SiC surface. (a) Cross-sectional HRTEM image acquired near the bulk region of the smooth laser-irradiated surface, revealing well-ordered atomic planes with no observable lattice distortion. (b) Cross-sectional HRTEM image obtained near the bulk region of the laser-sliced surface (hill area), showing similarly well-preserved crystallinity. The insets in (a) and (b) display the corresponding selected-area electron diffraction (SAED) patterns indexed along the [1-100] zone axis, confirming the identical crystal orientation and the absence of polytype transformation and defect generation induced by the laser processing.

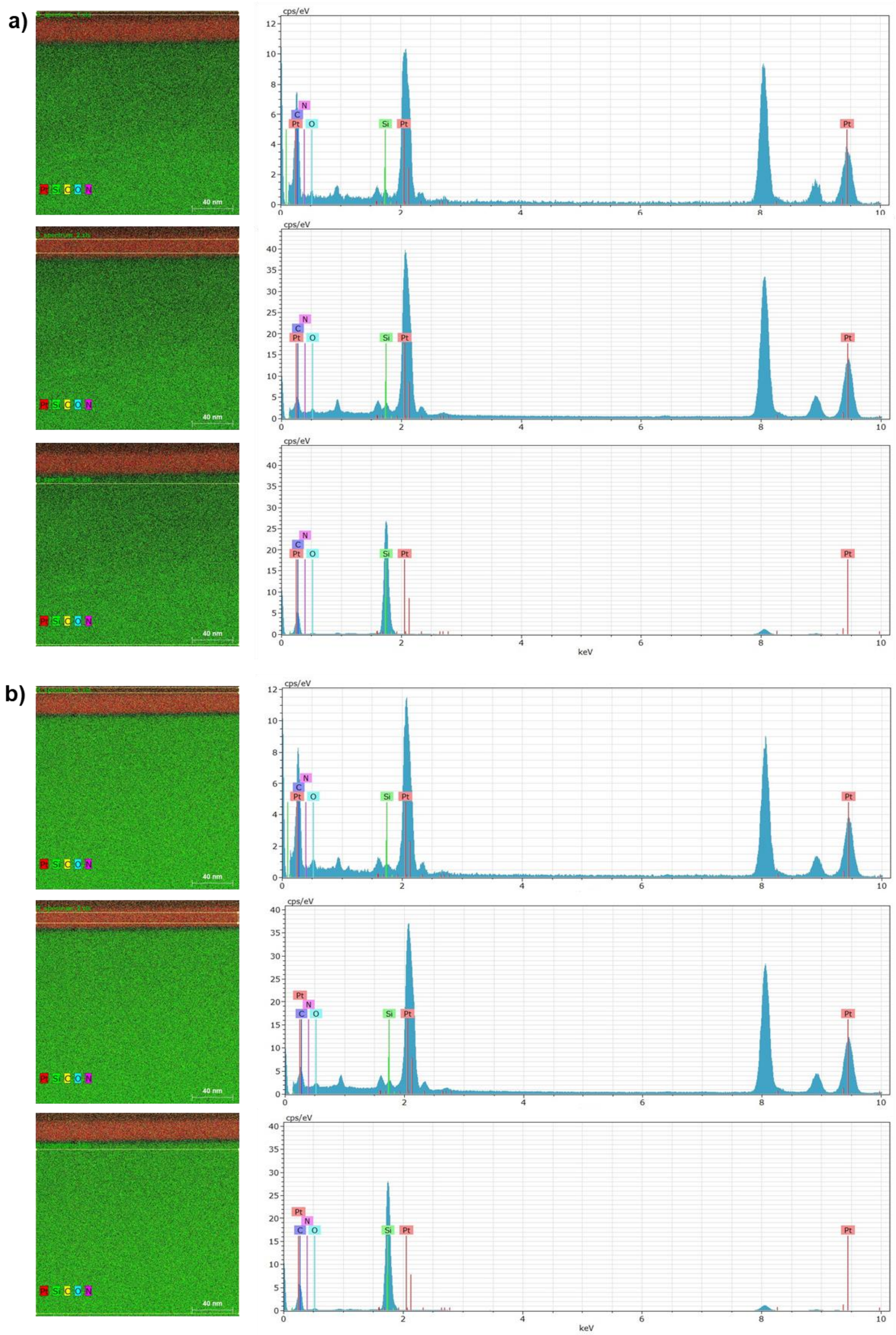

Figure S9. STEM–EDS maps and spectra for the 4H-SiC wafer. (a) Laser-irradiated surface. Left: HAADF image with overlaid elemental maps of Pt, Si, C, O and N. The analyzed areas are indicated by yellow rectangular outlines. Right: representative EDS spectra acquired at three positions from the near-surface region towards the bulk (top to bottom). The spectrum from the near-surface band immediately beneath the Pt cap shows a clearly enhanced O K_{α}

peak and slightly higher C intensity compared with the deeper regions, whereas the bulk spectrum is dominated by Si and C with only a very weak O signal. Pt peaks originate from the protective cap layer. (b) Laser-sliced surface. Left: HAADF image with overlaid elemental maps of Pt, Si, C, O and N. The analyzed areas are indicated by yellow rectangular outlines. Right: spectra from three positions analogous to Figure S9a. Again, the near-surface region exhibits a stronger O K_{α} peak than the deeper regions, while the bulk spectrum shows mainly Si and C with only trace oxygen, confirming that oxygen is localized in a thin band close to the surface whereas the underlying SiC remains nearly stoichiometric.

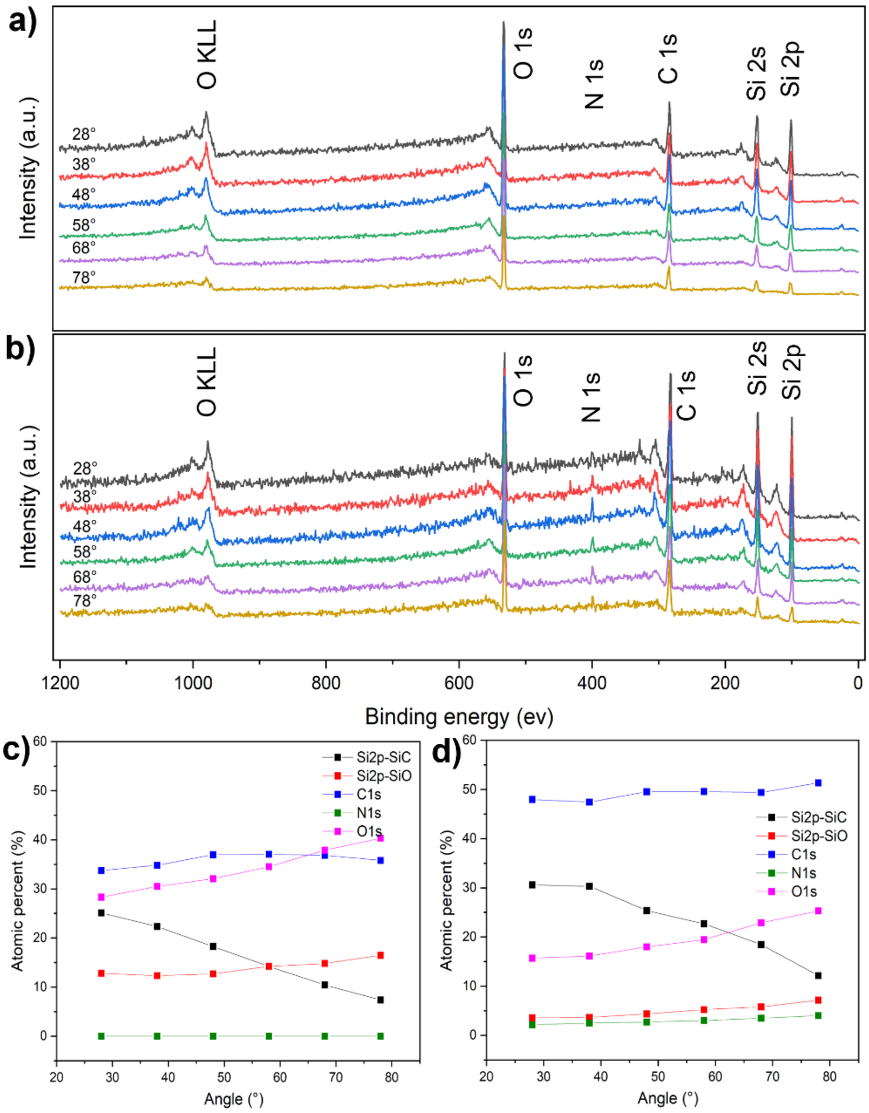

Figure S10. ARXPS survey spectra obtained from the two SiC surface and the angular dependence of the atomic percentages for Si2p–SiC, Si2p–SiO, C 1s, N 1s, and O 1s extracted from the two SiC surface. (a,b) ARXPS survey spectra obtained from the laser-irradiated surface (a) and the laser-sliced surface (b) at take-off angles ranging from 28° to 78°. The take-off angle is defined with respect to the surface normal (0°), such that larger angles provide increased surface sensitivity. Distinct core-level and Auger features, including O KLL, O 1s, N 1s, C 1s, Si 2s, and Si 2p, are observed for all angles, allowing evaluation of the depth-dependent chemical states. (c,d) Angular dependence of the atomic percentages for Si2p–SiC, Si2p–SiO, C 1s, N 1s, and O 1s extracted from the laser-irradiated surface (c) and the laser-sliced surface (d). The increase in the O 1s and Si2p–SiO fractions at higher angles indicates the presence of a thin surface oxide layer, while the decreasing Si2p–SiC contribution reflects reduced sampling of the underlying SiC lattice at surface-sensitive geometries. These trends collectively confirm that both surfaces exhibit a modified near-surface region confined to the top few nanometers, with the bulk SiC chemistry remaining intact beneath.

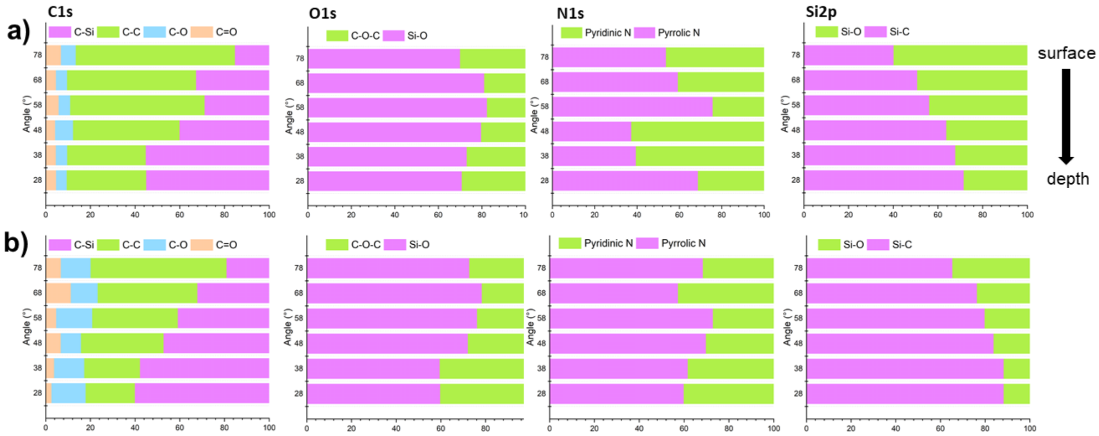

Figure S11. Relative atomic percentages of the deconvoluted chemical components obtained from the two SiC surfaces at take-off angles ranging from 28° to 78°. (a,b) Relative atomic percentages of the deconvoluted chemical components obtained from the laser-irradiated surface (a) and the laser-sliced surface (b) at take-off angles ranging from 28° to 78°. The deconvoluted species include C 1s components (C–Si, C–C, C–O, and C=O), O 1s components (C–O–C and Si–O), N 1s components (pyridinic N and pyrrolic N), and Si 2p components (Si–O and Si–C). The angular dependence of these species provides depth-sensitive information about the distributions of carbon bonding states, oxygen-containing surface groups, nitrogen configurations, and silicon oxidation states, enabling a comparative evaluation of the near-surface chemical structures of the laser-irradiated and laser-sliced SiC surfaces.

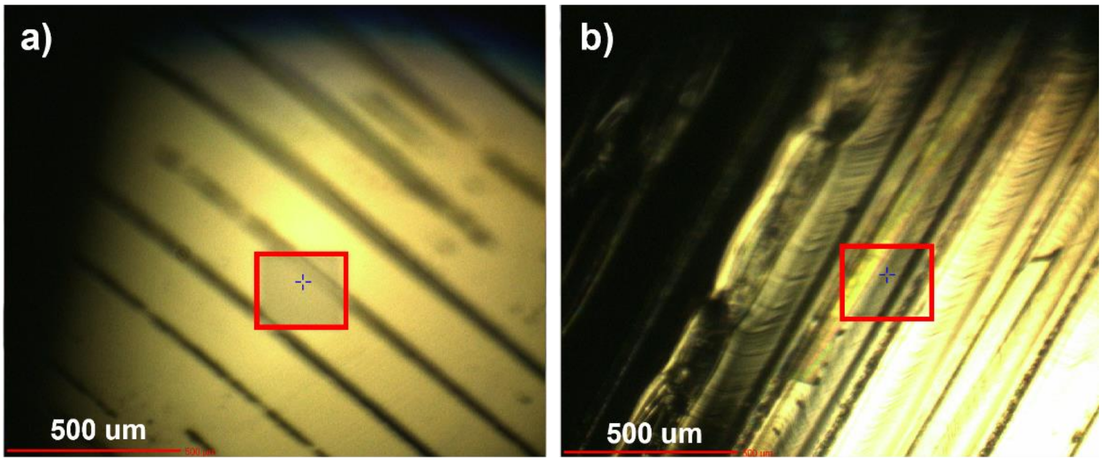

Figure S12. Optical microscopy images of the ion-etched craters formed during the SIMS depth profiling measurements on the two SiC surface. (a,b) Optical microscopy images of the ion-etched craters formed during the SIMS depth profiling measurements on the laser-irradiated surface (a) and the laser-sliced surface (b). The regions marked by red boxes indicate the areas where the ion beam was rastered to produce the analysis crater. Surface morphology differences between the two regions, originating from laser-induced surface modification and slicing-induced fracture texture, are clearly visible, reflecting distinct sputtering responses during SIMS profiling.

Table S1. Key laser processing conditions employed in the present study and compared with representative parameters from earlier reports on laser processing of SiC. The key laser processing conditions employed in the present study including wavelength, pulse duration, repetition rate, pulse energy, spot size, scan speed, fluence, ambient environment, and scan pattern are compared with representative parameters from earlier laser-processing reports on SiC. The comparison highlights differences in the irradiation wavelength (UV to NIR), pulse width (ps to ns), scanning strategies, processing atmospheres, and fluence regimes, providing context for evaluating the unique processing window and material response achieved in this work.

	Wavelength (λ)	Pulse duration (τ)	Repetition rate (f)	Pulse energy (E)	Spot diameter	Scan speed (v)	Fluence	Atmosphere	Polarization
This work	365 nm	10 ps	100 kHz	0.30 μJ	~8 μm	40 mm/s	1.2 J/cm²	N_2, 5–10 L/min	Linear
[1]	1030 nm	30 ps	100-200 kHz	1.3–2.1 W (laser power)	4 μm (Δd = 1.50 μm.)	200 mm/s	2.97–11.88 J/cm²	-	-
[2]	1552 nm	1, 5, 10 ps	200 kHz	5 mJ	-	10 mm/s	-	-	Nonlinear optical effect
[3]	1064 nm	12.6 ps	50 kHz (for initial test), 100 kHz (etching experiments)	1 mJ @ 10 kHz	1.22 λ (NA 2.88 μm)	100, 150, 200 mm/s	-	-	Linear
[4]	1070 nm	-	-	(Laser energy, 0 → 230 W)	1 mm	3 mm/min	-	-	-
[5]	351 nm	90 ns	1 kHz	-	350 μm	200–350 mm/s	6, 12 J/cm²	Air	-
[6]	1030 nm	3 ps	~1.83 MHz	25 μJ	17–20 μm	10 mm/s	-	Air	Linear
[7]	532 nm	232~693 ns	1~38 kHz	4.4, 16.3 mJ	~70 μm	10, 40, 100 mm/s	-	Air	Linear
[8]	1064 nm	25 ns	10 kHz	-	10 μm	10 mm/s to 30 mm/s	2.2, 38.9 J/cm²	-	3D XYZ stage
[9]	355 nm	28 nm @ 100 kHz	1~300 kHz	60, 120 μJ (Laser energy, 10W)	30 μm	0–20 mm/s	-	Air	-
[10]	1064 nm	4 ns	60 kHz	Average power 1.5 W	3 μm	200 mm/s	-	-	-
[11]	1064 nm	4 ns	80 kHz	Average power 3.2 W	10 μm	-	-	-	-
[12]	1064 nm	4 ns	80 kHz	Average power 3.4 W	3 μm	120–260 mm/s	-	-	-

Table S2. Elemental compositions obtained from EDS measurements performed on the two SiC surface. Elemental compositions obtained from EDS measurements performed on both the laser-irradiated surface and the laser-sliced surface of the 4H-SiC wafer. The data compare the relative concentrations of Si, C, and O detected from each region, allowing evaluation of laser-induced surface modifications, including oxygen incorporation and changes in the Si/C ratio associated with irradiation and slicing processes.

	Element	wt%	Atomic %
Laser-irradiated surface	C	33.88	54.14
	O	1.29	1.55
	Si	64.83	44.31
	Total	100.00	100.00
Laser-sliced surface	C	35.49	56.26
	Si	64.51	43.74
	Total	100.00	100.00

Table S3. Detailed fitting of the deconvoluted XPS peaks obtained from the laser-irradiated SiC surface. The parameters extracted by the fitting include peak positions, full width at half maximum (FWHM), and the relative atomic percentages of the individual chemical states associated with the C 1s, O 1s, N 1s, and Si 2p core levels. These values provide quantitative insight into the bonding environments and surface chemical modifications induced by laser irradiation.

	C 1s				O 1s		N 1s		Si 2p	
**Chemical bonding**	**C–Si**	**C–C/C-H**	**C–O**	**C=O**	**Si–O**	**C–O–C**	**Pyridinic N**	**Pyrrolic N**	**Si–O**	**Si–C**
Peak position (B.E. eV)	282.6	284.6	286.0	288.0	532.5	531.5	398.9	400.3	102	100
**28°**										
**FWHM of peak (eV)**	0.90	2.01	1.14	2.62	1.82	2.75	1.61	2.72	3.5	1.83
**Concentration (mol%)**	55.0	35.6	4.8	4.6	70.8	29.2	1.8	98.2	28.5	71.5
**38°**										
**FWHM of peak (eV)**	0.94	2.00	1.30	2.54	1.82	3.03	3.43	0.56	2.03	1.84
**Concentration (mol%)**	55.2	35.2	5.1	4.5	73.0	27.0	60.6	39.4	32.3	67.7
**48°**										
**FWHM of peak (eV)**	1.00	2.14	1.18	2.22	1.75	3.01	3.49	1.45	1.96	1.83
**Concentration (mol%)**	40.1	47.7	8.0	4.2	79.6	20.4	62.7	37.3	36.3	63.7
**58°**										
**FWHM of peak (eV)**	1.17	2.20	1.16	2.38	1.85	3.15	1.37	1.19	2.02	1.89
**Concentration (mol%)**	28.9	60.2	5.2	5.7	82.3	17.7	24.3	75.7	43.9	56.1
**68°**										
**FWHM of peak (eV)**	1.07	1.92	0.96	2.34	1.57	1.59	3.35	1.72	1.96	2.08
**Concentration (mol%)**	32.7	57.8	4.9	4.6	81.0	19.0	40.7	59.3	49.3	50.7
**78°**										
**FWHM of peak (eV)**	1.39	2.00	1.02	2.35	1.69	2.87	3.48	1.67	2.05	1.98
**Concentration (mol%)**	15.3	71.2	6.7	6.8	70.0	30.0	46.3	53.7	59.9	40.1

Table S4. Detailed fitting of the deconvoluted XPS peaks obtained from the laser-sliced SiC surface. The parameters extracted by the fitting include peak positions, full width at half maximum (FWHM), and the relative atomic percentages of the individual chemical states associated with the C 1s, O 1s, N 1s, and Si 2p core levels. These values provide quantitative insight into the bonding environments and surface chemical modifications induced by laser slicing.

	C 1s				O 1s		N 1s		Si 2p	
**Chemical bonding**	**C–Si**	**C–C/C-H**	**C–O**	**C=O**	**Si–O**	**C–O–C**	**Pyridinic N**	**Pyrrolic N**	**Si–O**	**Si–C**
Peak position (B.E. eV)	282.6	284.6	286.0	288.0	532.5	531.5	398.9	400.3	102	100
**28°**										
**FWHM of peak (eV)**	1.00	1.37	2.11	1.16	1.84	2.08	3.5	1.46	1.67	1.29
**Concentration (mol%)**	60.1	22	15.3	2.6	59.7	40.3	40.2	59.8	11.7	88.3
**38°**										
**FWHM of peak (eV)**	1.44	1.47	2.13	1.2	1.83	2.30	3.48	1.34	1.85	1.30
**Concentration (mol%)**	57.8	25	13.5	3.7	59.5	40.5	38.4	61.6	11.7	88.3
**48°**										
**FWHM of peak (eV)**	1.08	1.73	1.78	1.55	1.91	2.45	3.31	1.45	2.10	1.23
**Concentration (mol%)**	47.2	37	9.1	6.7	72.1	27.9	30.2	69.8	16.2	83.8
**58°**										
**FWHM of peak (eV)**	1.05	1.63	1.70	1.28	1.73	1.89	1.94	1.21	2.13	1.19
**Concentration (mol%)**	40.9	38.4	16.1	4.7	76.2	23.8	27.1	72.9	20.2	79.8
**68°**										
**FWHM of peak (eV)**	0.85	1.58	1.32	1.37	1.94	2.33	3.49	1.45	1.92	1.24
**Concentration (mol%)**	32.1	44.6	12.1	11.2	78.3	21.7	42.7	57.3	23.7	76.3
**78°**										
**FWHM of peak (eV)**	1.10	1.50	1.60	1.33	1.78	1.98	2.60	1.43	2.01	1.22
**Concentration (mol%)**	19.1	60.8	13.4	6.8	72.7	27.3	31.6	68.4	34.6	65.4

References

[1] Liu, F.; Xu, J.; Yan, S.; Zhou, Y.; Zhang, Y. Mechanism and regulation of thermal damage on picosecond laser modification dicing of SiC wafer. *Chemical Engineering Journal.* **2024**, 493.

[2] Yang, B.; Wang, H.; Peng, S.; Cao, Q. Precision layered stealth dicing of SiC wafers by ultrafast lasers. *Micromachines (Basel).* **2022**, 13 (7).

[3] Han, S.; Yu, H; He, C.; Zhao, S.; Ning, C.; Jiang, L.; Lin, X. Laser slicing of 4H-SiC wafers based on picosecond laser-induced micro-explosion via multiphoton processes. *Optics & Laser Technology.* **2022**, 154.

[4] Cao, C.; Zhao, Y.; Zhang, G.; Li, Z.; Zhao, C.; Yu, H.; Zhao, D.; Zhang, H.; Dai, D. Experimental study of plastic cutting in laser-assisted machining of SiC ceramics. *Optics & Laser Technology.* **2024**, 169.

[5] Zhang, L.; Zhu, Y.; Yang, K.; Zhou, J.; Chen, J. Study on surface modification of 4H-SiC wafers induced by nanosecond laser. *Optics & Laser Technology.* 2025, 192.

[6] Amsellem, W.; Sarvestani, H.Y.; Pankov, V.; Martinez-Rubi, Y.; Gholipour, J.; Ashrafi, B. Deep precision machining of SiC ceramics by picosecond laser ablation. *Ceramics International.* **2023**, 49, 9592–9606.

[7] Elkington, H.; Marimuthu, S.; Smith, B. High power water jet guided laser cutting of SiC/SiC ceramic matrix composite. *Journal of Laser Micro/Nanoengineering.* **2022**, 17, 168–173.

[8] Xiangfu, L.; Minghui, H. Micro-cracks generation and growth manipulation by all-laser processing for low kerf-loss and high surface quality SiC slicing. *Optics Express.* **2024**, 32, 38758–38767.

[9] Tseng, S.-F.; Luo, C.-X.; Hsiao, W.-T. Characterization analysis of 355 nm pulsed laser cutting of 6H-SiC. *The International Journal of Advanced Manufacturing Technology.* **2023**, 130, 3133–3147.

[10] Hirata, K., and R. Yamamoto, Method for producing SiC wafer. *Korean Patent*, **2018**. KR 10-2018-0094798 A.

[11] Suzuki, K., Method of machining SiC wafer. *Korean Patent*, **2020**. KR 10-2174875 B1.

[12] Hirata, K., and Y. Morishige, SiC wafer producing method. *US Patent*, **2018**. US 9,878,397 B2.
